# Passive knee flexion increases forward impulse of the trailing leg during the step-to-step transition

**DOI:** 10.1038/s41598-025-95589-4

**Published:** 2025-04-15

**Authors:** Bernadett Kiss, Alexandra Buchmann, Daniel Renjewski, Alexander Badri-Spröwitz

**Affiliations:** 1 Max Planck Institute for Intelligent Systems, Stuttgart, 70569 Germany; 2 Chair of Applied Mechanics, TUM School of Engineering & Design, Department of Mechanical Engineering, Technical University of Munich, Garching near Munich, 85748 Germany; 3 Department of Mechanical Engineering, KU Leuven, Leuven, 3000 Belgium

**Keywords:** Human walking, Catapult mechanism, Push-off, Knee flexion initiation, Gait event timing, Bipedal robot, Mechanical engineering, Bone quality and biomechanics, Biomimetics, Rehabilitation

## Abstract

During walking, the brain and nervous system coordinate muscle activity to efficiently regulate body movement. Simultaneously, passive structures in the legs interact with the ground, generating reaction forces that contribute to leg and body motion. A well-known example of this active-passive coordination is the human ankle, which plays a crucial role in propelling both the leg and the entire body forward with each step. Human walking efficiency relies on the elastic recoil of the Achilles tendon, facilitated by a “catapult mechanism” that stores energy during stance and releases it during push-off. The catapult release mechanism could include the passive flexion of the knee, as the main part of knee flexion was reported to happen passively after leading leg touch-down. This study is the first to investigate the effects of passive versus active knee flexion initiation, using the bipedal EcoWalker-2 robot with passive ankles. By leveraging the precision of robotic measurements, this study aimed to elucidate the importance of timing of gait events and its impact on momentum and kinetic energy changes of the robot. The EcoWalker-2 walked successfully with both initiation methods, maintaining toe clearance. Passive knee flexion initiation delayed the onset of ankle plantar flexion by 3% of the gait cycle compared to active knee flexion initiation, leading to 87% larger increase in the trailing leg horizontal momentum, and 188% larger magnitude increase in the center of mass momentum vector during the step-to-step transition. The findings highlight the role of knee flexion in the release of the catapult and timing of gait events. These insights contribute to improving the control and mechanics of human-centered robotic and assistive devices. Specifically, enabling passive knee flexion initiation could be beneficial in humanoid robots with passive ankles, and in ankle-knee prostheses and orthoses with passive ankles for saving on control effort, and reducing hardware complexity otherwise required for active knee flexion before the step-to-step transition. Additionally, this approach enhances horizontal momentum gain in the trailing leg during the step-to-step transition, with the potential to improve locomotion efficiency.

## Introduction

Human walking gait emerges from the intricate interplay between neural motor control, reflexes, muscle dynamics, and passive structures. Notably, ankle mechanics play a crucial role, with the ankle plantar flexor muscles contributing up to 78% of the total positive muscle work during push-off^1^. Moreover, the elastic recoil of the mechanically passive Achilles tendon is responsible for up to 91% of the ankle power output during push-off^2^. A catapult mechanism in the human leg was proposed to generate the high power output at the ankle by gradually storing energy during stance and then quickly releasing the energy during push-off^3,4^. A possible catapult release mechanism could include the passive flexion of the knee joint, as the main part of knee flexion was reported to happen passively after leading leg touch-down^5^. Building on Perry’s observation, this study investigates the effects of passive versus active knee flexion initiation on gait events around push-off using a robotic system. By leveraging the precision of robotic measurements, this study aims to elucidate the importance of timing of gait events and its impact on momentum and kinetic energy changes in the trailing leg, remaining body, and center of mass. Understanding the biological mechanisms driving natural leg dynamics during push-off is essential for advancing gait rehabilitation^6^, prosthesis^7^, orthosis, and exoskeleton development^8^, as well as efficient legged robot design^9^.

The principles of passive dynamics underlying human gait have been extensively investigated with robots^10,11^. However, none of the passively walking bipedal robots based on passive dynamics have been studying the human leg catapult function specifically. Instead, they were designed with other goals in mind; the Cornell biped was designed for minimal cost of transport, the MIT learning biped was designed to test motor learning utility on a passive-dynamic mechanical design, and the Delft biped was designed to test skateboard-like ankle joints for lateral stability^11^.

The EcoWalker-2 robot, a new version of the EcoWalker robot^12^, in contrast to the crouched gait of many humanoids is capable of straight-leg walking gait. Straight-leg walking is more human-like and it improves energy efficiency as the required torque and energy consumption to support the body weight at the knee joints is lower than during bent-knee walking^13,14^. A robot walking with straight legs in midstance can achieve greater ground clearance, enabling it to navigate over larger obstacles and reduce the risk of collisions, while also reducing the necessary range of motion and actuator velocities required to maintain a given walking speed^15^.

To achieve efficient walking, the EcoWalker-2 robot draws inspiration from the human leg’s unique mechanical properties. In particular, the human leg can be thought of as a catapult system, where elastic energy is stored and rapidly released to facilitate movement. A catapult has three main mechanical components: an elastic element, a block, and a catch. In the human leg, it is challenging to identify the specific catapult mechanics and their functional elements due to the complex interplay between the thigh-shank-foot segment chain and muscle-tendon units at the ankle joint. Elastic energy storage and rapid recoil during ankle push-off are facilitated by the Achilles tendon, which is attached to the Soleus (SOL) and Gastrocnemius (GAS) muscles^18–21^. The GAS muscle’s mostly isometric work facilitates energy-efficient loading of the Achilles tendon, while the SOL muscle contributes to ankle coordination through active contraction^22^. The EcoWalker robot was used to demonstrate the benefits of a SOL spring-tendon, which amplified ankle power and enabled more efficient and faster walking^12^. In contrast, a GAS spring-tendon was shown to modify the coordination between the ankle and knee joints during ankle push-off^12^. The block, serving as the supporting structure of the catapult, was identified to be a combination of the foot and the ground^23^. The catch, a crucial component that locks and releases the human leg catapult, remains unidentified.

During the release of the catapult, the knee and the hip start to flex, and the ankle starts to plantarflex. A possible release mechanism can be derived from Perry’s work^5^. The main part of knee flexion, from 5$^{\circ }$ to 40$^{\circ }$, was reported to happen passively after leading leg touch-down, as weight transfer to the leading leg frees the trailing leg’s knee joint to flex passively^5^ (p. 104f).

In this work and for the first time, Perry’s passive knee flexion process was tested on a bipedal robot, as part of the lock-and-release mechanism of the human leg catapult. This study investigates whether passive knee flexion resulting from leg dynamics can trigger the human leg catapult release on a bipedal robot. The same robotic system, the EcoWalker-2 robot^12^, was compared with and without active knee flexion initiation: active knee flexion initiation (AKFI) versus passive knee flexion initiation (PKFI).

In the PKFI experiment, the trailing leg’s knee motor does not apply torque in the EcoWalker-2 robot^12^, the knee joint can move freely, from midstance until the next touch-down of the trailing leg. In the meantime, passive SOL and GAS spring-tendons provide plantar flexion and knee flexion moments. For sufficient toe clearance, knee flexion must happen before the start of the swing. As the knee motors will not provide knee flexion moment in the PKFI experiment, knee flexion is expected to be a result of knee flexion moment from the GAS spring-tendon and knee flexion moment from the ground reaction force.

**Fig. 1:**
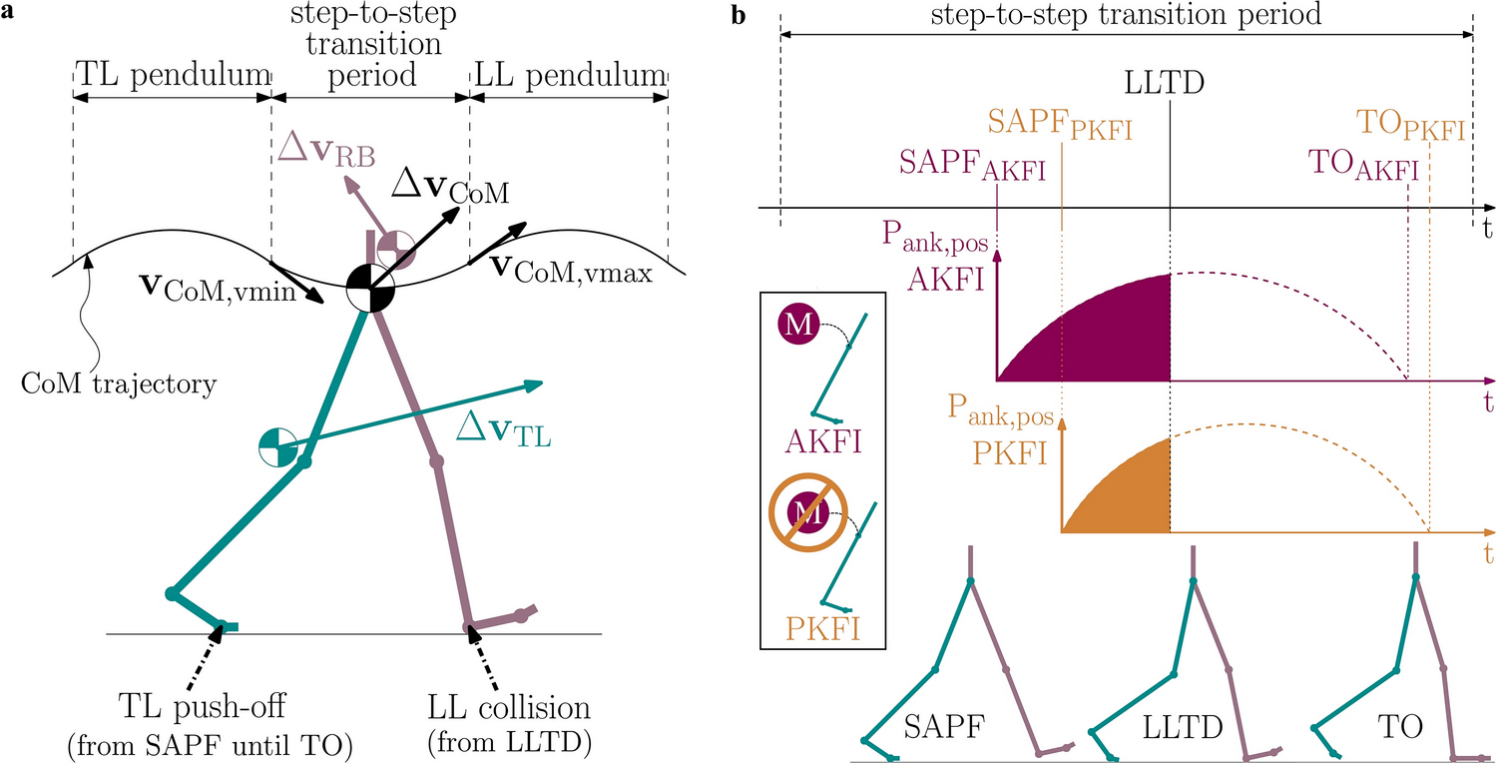
The two main roles of push-off (**a**) and the expected effect of passive versus active knee flexion initiation (**b**). The main part of knee flexion, from 5-40deg, occurs passively after LLTD^5^. This study compares PKFI and AKFI to influence energy flow into the RB and TL by indirectly manipulating SAPF timing. This study aims to contribute to the debate on the primary function of ankle push-off in the human swing leg catapult mechanism^16^. (**a**) During a step-to-step transition, push-off plays two main roles: it redirects the center of mass velocity ($\Delta {\textbf{v}}_\textrm{CoM}$) from one inverted pendulum arc to the next, and it increases the velocity of the TL. The drawing was inspired by Fig. 1 in Ref.^17^ and Fig. 4 in Ref.^4^. (**b**) The AKFI experiment was designed with earlier knee flexion onset than the PKFI experiment to alter the SAPF timing. This results in an earlier SAPF in the AKFI experiment, prolonging the positive ankle power ($\textrm{P}_\textrm{ank, pos}$) period until LLTD. Before LLTD, the TL bears the whole body weight, while the loading leg (LL) is in swing. The filled part under the positive ankle power curve represents the predicted ankle energy mainly accelerating the RB. Due to the predicted SAPF delay in the PKFI experiment, lower ankle energy could accelerate the RB in the PKFI than in the AKFI experiment. TL: Trailing Leg, LL: Leading Leg, RB: Remaining Body, CoM: Center of Mass, vmin: time of minimum vertical velocity of the CoM (start of Step-to-Step transition period), vmax: second vertical velocity peak of the CoM after vmin (end of Step-to-Step transition period), SAPF: Start of Ankle Plantar Flexion, LLTD: Leading Leg Touch-Down, TO: Toe-Off, AKFI: Active Knee Flexion Initiation, PKFI: Passive Knee Flexion Initiation.

As knee flexion induces ankle alleviation^4^, it enables the onset of ankle plantarflexion. In the AKFI experiment, knee flexion onset timing is expected to influence the timing of the start of ankle plantar flexion. The start of ankle plantar flexion is influenced by the active hip and active knee flexion that pull the foot off the ground during the AKFI experiment. At the start of ankle plantar flexion the ankle power becomes positive, indicating the start of ankle unloading. Ankle unloading, the positive ankle power period, lasts until toe-off (Fig. 1b).

In the AKFI experiment, knee flexion is actively initiated as the knee motors are commanded to follow a predefined trajectory during the whole gait cycle. The onset of knee flexion is defined in the desired knee angle curve earlier than in the PKFI experiment. Due to the earlier timing of knee flexion onset, the start of ankle plantar flexion is also expected earlier in the AKFI experiment than in the PKFI experiment (Fig. 1b).

To reach a symmetric gait with the bipedal EcoWalker-2 robot, the phase shift in the control of the hip motors is a constant 50% of the gait cycle. As the phase shift is constant, the time of leading leg touch-down relative to trailing leg touch-down is not expected to largely change between the AKFI and PKFI experiments. Leading leg touch-down starts the weight transfer from the trailing leg to the leading leg, the trailing leg is freed up to go into swing during the double support phase.

Consequently, the time period between the start of ankle plantar flexion and leading leg touch-down is expected to be shorter in the PKFI experiment than in the AKFI experiment (Fig. 1b).

Accurately studying the effects of such a gait event timing change can be challenging due to the inherent variability in human gait data, where the average standard deviation of gait events can be as high as 1.5% of the gait cycle^24^. In contrast, a robotic system can demonstrate greater repeatability, with an average standard deviation of less than 0.2% of the gait cycle for gait events (see Supplementary Table S6). The almost tenfold better precision of robotic measurements allowed us to investigate the crucial role of timing of gait events and its influence on momentum and kinetic energy changes in the trailing leg, remaining body, and center of mass.

This investigation into the effects of timing of gait events has wide-ranging implications for understanding the main function of push-off, a topic of ongoing debate in the field. Some researchers (*leg swing group*) argue that push-off energy primarily accelerates the swing leg, while others (*redirection group*) propose that it aids in redirecting the center of mass velocity to reduce collision losses^16^ (Fig. 1a). However, despite the extensive discussion on the function of push-off, the influence of gait event timing on the function of push-off has remained largely unexplored. Examining the factors that influence the push-off energy flow, such as the timing of ankle plantar flexion, can provide new insights into the function of push-off in gait and contribute to resolving the long-standing debate.

The *leg swing group* of researchers see the main role of ankle push-off in its contribution to leg swing acceleration^1,4,25,26^ (Fig. 1). Segmental energy flow analysis on human walking data showed that the increase in the trailing leg energy is 90% of the positive ankle energy generated by the plantarflexor muscles during push-off^1^. Meanwhile the energy increase of the trunk during push-off is 13% of the positive ankle push-off energy^1^.

In human walking, each step-to-step transition involves the redirection of the center of mass (CoM) velocity from one inverted pendulum arc to the next^17,27^ (Fig. 1a). The step-to-step transition period, defined as the interval from minimum to maximum vertical center of mass velocity, was used to capture the effect of the transition from trailing leg single support to leading leg single support^17^ (Fig. 1). The *redirection group* of researchers suggest that, during the step-to-step transition, the main function of ankle push-off is aiding the redirection of the CoM velocity^17,27–35^. Experimental human gait data at 0.9m/s to 1.8m/s walking velocities shows that CoM energy change during push-off is 86%-96% of ankle push-off energy^36^. The *redirection group* simulated human gait with simple models with or without a knee joint, and considered the body as a whole. The physics-based models predict that collision losses can be reduced by pushing off before leading leg touch-down^17,27–29,33,37,38^. However, Donelan et al. point out that their calculation method cannot differentiate between the effects of swing leg motion and CoM redirection^27,29^.

The combined idea of both *redirection group* and *leg swing group* were already considered in biomechanics research in 1966^39^. Later Zelik and Adamczyk^16^ supported the view that ankle push-off propels both the trailing leg into swing and the body over the leading leg. The ankle push-off energy contributes to leg swing acceleration, i.e. kinetic energy increases in the swing leg, but only a small part of the kinetic energy is transferred to the torso via the hip^1,4,16^. However, as the swing leg is part of the CoM calculations, the swing leg acceleration does increase the CoM velocity, so most of the kinetic energy change in the swing leg also appears as a CoM kinetic energy change^16^ (Fig. 1a). Therefore, Zelik and Adamczyk^16^ suggest that the consideration of ankle mechanics should avoid the binary contrast of leg swing versus CoM redirection. Instead, in the interpretation of the function of the ankle push-off Zelik and Adamczyk^16^ propose taking into account both swing leg acceleration and CoM redirection effects simultaneously.

To further illustrate this concept, let us consider the effect of trailing leg velocity change on the whole body CoM velocity change. By modeling the body as a system of segments, divided into the trailing leg and the remaining body, the contribution of each segment to the whole body CoM momentum can be calculated. The remaining body consists of the leading leg and the head-arms-trunk segments (Fig. 1a). Notably, the trailing leg’s contribution to the whole body CoM momentum can be substantial, despite its smaller mass, if its velocity is high^4,16^. This is evident in experimental human gait data at the preferred transition speed between walking and running, where the trailing leg’s horizontal momentum increased by 22.2Ns during push-off, while the remaining body’s horizontal momentum decreased by 7.8Ns. Although the trailing leg’s mass (11.4kg) is only about one-sixth that of the remaining body (59.5kg), its large velocity change resulted in a net increase in the whole body CoM’s horizontal momentum by 14.4Ns^4^.

During push-off, the momentum changes of the trailing leg and the remaining body depend on how much of the ankle push-off energy is transferred through the hip. Energy transfer during push-off could be influenced by the body weight distribution between the trailing and the leading leg. Specifically, when the trailing leg bears the body’s weight during the single support phase, larger proportion of the ankle energy in the trailing leg could contribute to accelerating the rest of the body. As the leading leg touches down and becomes the weight-bearing leg, the trailing leg’s ankle energy may increasingly accelerate the trailing leg into its swing phase, rather than the rest of the body (Fig. 1b). Hence, the exact timing of the start of ankle plantar flexion and the leading leg touch-down could largely alter the step-to-step transition dynamics.

In the AKFI experiment, the longer duration between the start of ankle plantar flexion and leading leg touch-down is expected to result in a greater proportion of ankle energy being transferred to accelerate the remaining body, and a lesser proportion being used to accelerate the trailing leg during the step-to-step transition period, compared to the PKFI experiment (Fig. 1b). It is hypothesized that the momentum of the remaining body $({\textbf {p}}_\text {RB})$ increases more and the momentum of the trailing leg (${\textbf {p}}_\text {TL}$) increases less during the step-to-step transition in the AKFI experiment compared to the PKFI experiment.

## Results

**Fig. 2:**
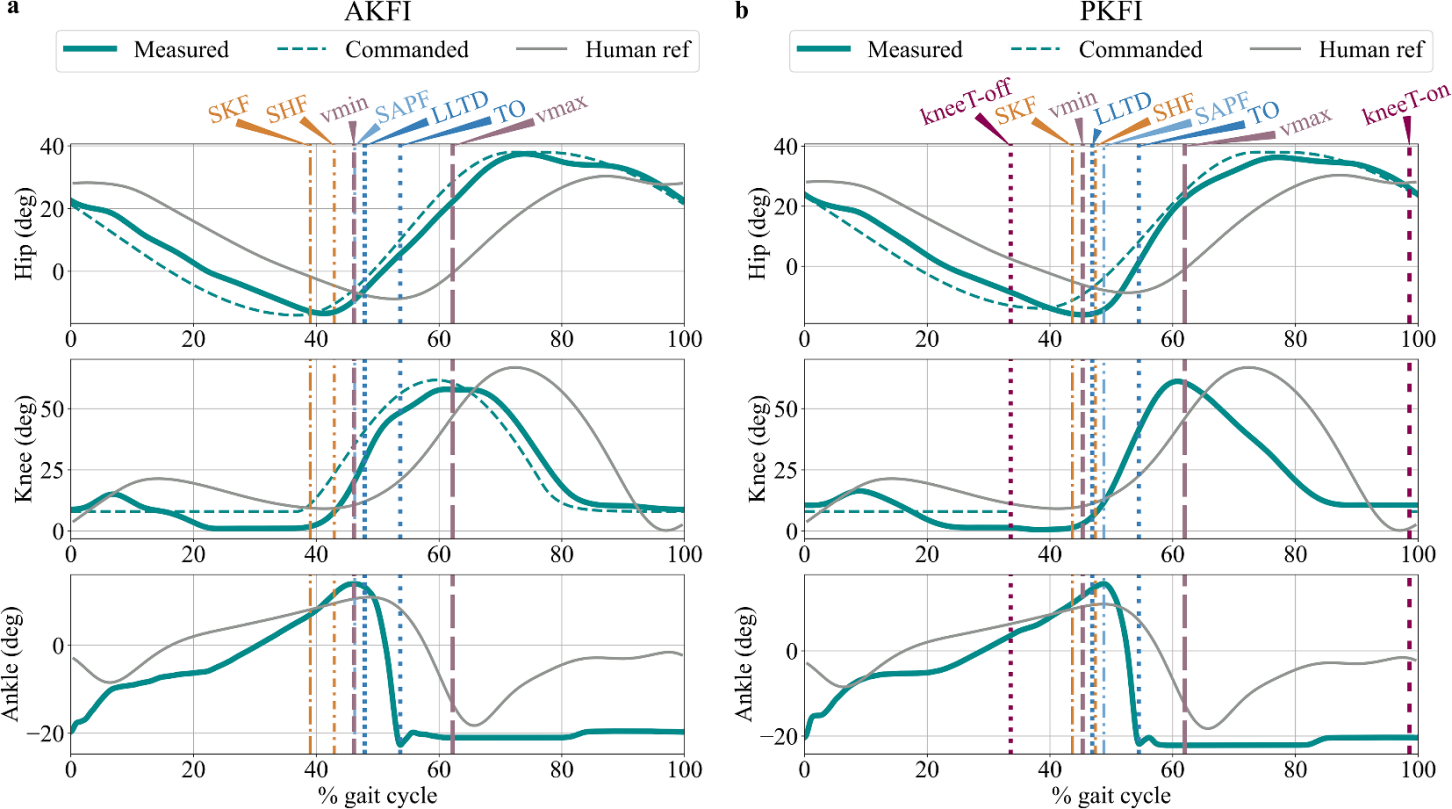
Hip, knee, and ankle angles during the full gait cycle in the experiments with active knee flexion initiation (AKFI - (**a**)), and with passive knee flexion initiation (PKFI - (**b**)). Shading shows the standard deviation of the curves. Horizontal axis shows the gait cycle (GC) percentage, 0% GC is the touch-down of the trailing leg. In the PKFI experiment, the knee motor torque is zero from the dark pink vertical dotted line (kneeT-off) until the dark pink vertical dashed line (kneeT-on). The continuous cyan line shows the measured joint angles of the robot, while the dashed cyan line shows the commanded joint angles of the hip and the knee. Joint angles of human walking are overlayed (gray lines) for reference.^40^$$^\text {: avg. of trials 20, 21, and 22}$$. SKF: Start of Knee Flexion, SHF: Start of Hip Flexion, SAPF: Start of Ankle Plantar Flexion, vmin: time of minimum vertical velocity of the center of mass (CoM), LLTD: Leading Leg Touch-Down, TO: Toe-Off, vmax: second vertical velocity peak of the center of mass (CoM) after vmin.

Similar hip, knee, and ankle joint angles were reached with active knee flexion initiation (AKFI) and with passive knee flexion initiation (PKFI) (Fig. 2). The robot could swing its leg with a flexed knee by passive knee flexion initiation (Fig. 2), allowing sufficient toe clearance during swing (see Supplementary Figs. S3 and S4, and Supplementary Movies S1 and S2). The robot reached an average walking speed of 0.44m/s with PKFI, the same as the average walking speed with AKFI.

**Fig. 3:**
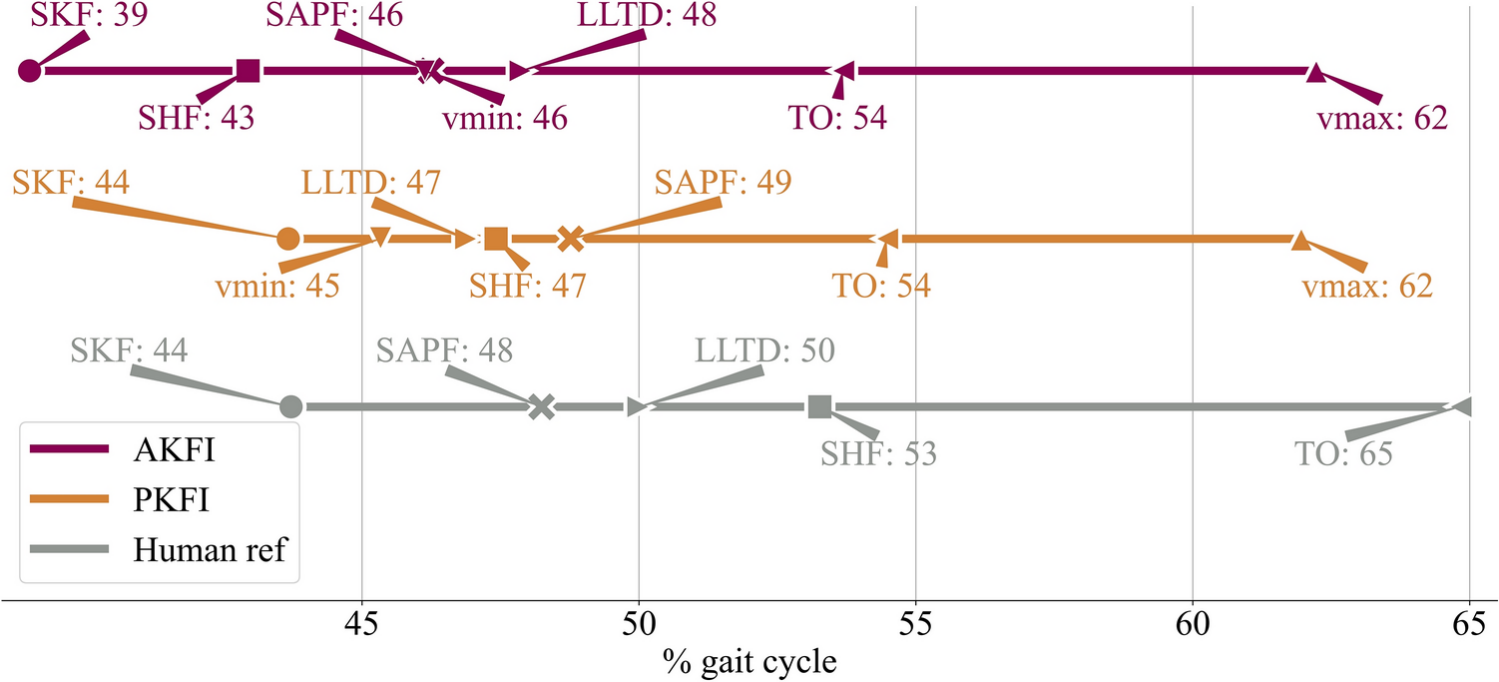
Timing of the gait events in gait cycle (GC) percentage with active knee flexion initiation (AKFI), and with passive knee flexion initiation (PKFI), and by humans^40^. 0% GC is the touch-down of the trailing leg. In the PKFI experiment, knee and hip flexion start 5% GC later than in the AKFI experiment (SKF and SHF). LLTD occurs 1%GC earlier with PKFI than with AKFI. The ankle starts to plantarflex (SAPF) 2% GC after LLTD with PKFI, while SAPF occurs 2% GC before LLTD with AKFI. The gait event timing values and their standard deviation values are available in Supplementary Table S2. $\bullet$ SKF: Start of Knee Flexion, $\blacksquare$ SHF: Start of Hip Flexion, $\times$ SAPF: Start of Ankle Plantar Flexion, $\blacktriangledown$ vmin: time of minimum vertical velocity of the center of mass (CoM), $\blacktriangleright$ LLTD: Leading Leg Touch-Down, $\blacktriangleleft$ TO: Toe-Off, $\blacktriangle$ vmax: second vertical velocity peak of the center of mass (CoM) after vmin.

A delay in the gait events can be observed in the PKFI experiment compared to the AKFI experiment (Fig. 3). Leading leg touch-down (LLTD) happens at 47% gait cycle (GC) and at 48% GC with PKFI and with AKFI, respectively. The knee joint starts to flex (Start of Knee Flexion - SKF) 5% GC later, the hip joints start to flex (Start of Hip Flexion - SHF) 4% GC later, and the ankle starts to plantarflex (Start of Ankle Plantar Flexion - SAPF) 3% GC later with PKFI than with AKFI (Fig. 3). The ankle starts to plantarflex (SAPF) 2% GC before leading leg touch-down (LLTD) with AKFI while the ankle plantarflexes (SAPF) only 2% GC after leading leg touch-down (LLTD) with PKFI (Fig. 3). The time where the ankle starts to plantarflex (SAPF) indicates when the springs spanning the ankle joint start to unload with positive ankle joint power profile (see Supplementary Fig. S9).

SKF occured significantly later (5%GC) in PKFI than in the AKFI experiment (p < 0.001) (Table 1, Fig. 3, and Supplementary Table S2). The time period between SAPF and LLTD is significantly different with p < 0.001 between the AKFI and PKFI experiment (Table 1, Fig. 3, and Supplementary Table S2).

**Fig. 4:**
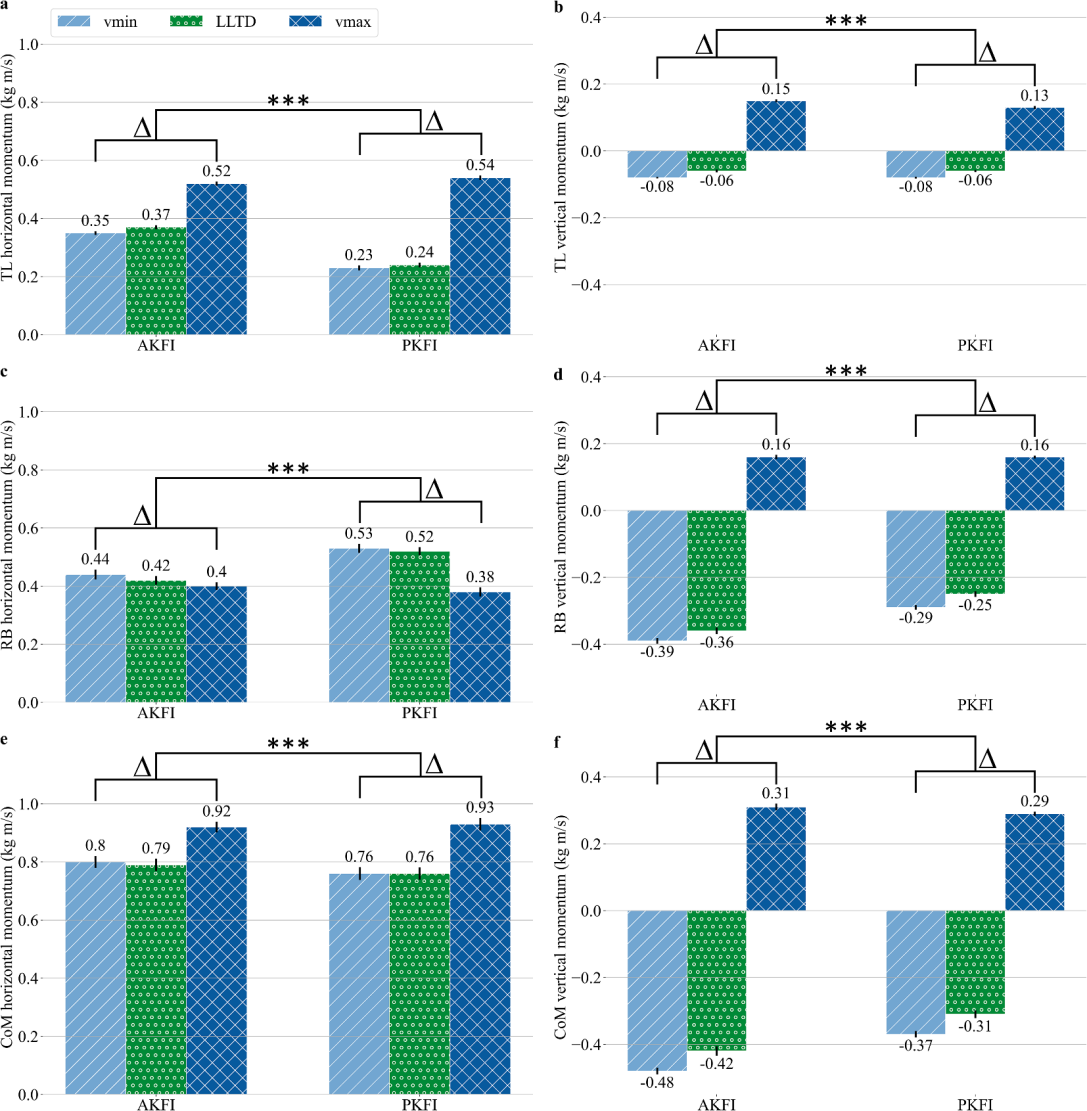
Trailing Leg (TL), Remaining Body (RB), and Center of Mass (CoM) instantaneous momentums in horizontal (**a**, **c**, and **e**) and vertical (**b**, **d**, and **f**) directions at the start of the step-to-step transition (vmin), at leading leg touch-down (LLTD), and at the end of the step-to-step transition (vmax) in the active knee flexion initiation (AKFI) and in the passive knee flexion initiation (PKFI) experiments. The vertical black lines at the top of the bars show the standard deviations. The change in TL horizontal momentum is larger with PKFI than with AKFI, and RB horizontal momentum decreases more with PKFI. The CoM’s vertical momentum increases more in AKFI than in the PKFI experiment during the step-to-step transition. The momentum values and their standard deviation values are available in Supplementary Table S3. *** denote significant difference between the momentum changes ($\Delta$) during the step-to-step transition period in the AKFI and in the PKFI experiments with p < 0.001. The exact p values are available in Table 1.

The vertical and horizontal momentum changes of the trailing leg and remaining body are significantly different between the AKFI and PKFI experiments Table 1. The instantaneous momentum of the trailing leg increases by 0.14kg m/s more in the horizontal direction with PKFI compared to AKFI during the step-to-step transition (Fig. 4a, Table 1, and Supplementary Fig. S5). The trailing leg’s momentum changes by 0.23kg m/s and by 0.21kg m/s in the vertical direction during the step-to-step transition in the AKFI and PKFI experiments, respectively (Fig. 4b, Table 1, and Supplementary Fig. S6). The horizontal momentum of the remaining body decreases by 0.15kg m/s with PKFI while it decreases by 0.04kg m/s with AKFI during the step-to-step transition (Fig. 4c, Table 1, and Supplementary Fig. S5). The remaining body’s vertical momentum increases 0.1kg m/s less with PKFI than with AKFI during the step-to-step transition (Fig. 4d, Table 1, and Supplementary Fig. S6).

**Table 1:** Results of the Wilcoxon signed-rank tests (p values) to test the differences between the active knee flexion initiation (AKFI) and the passive knee flexion initiation (PKFI) experiments.

Measure		p value	AKFI		PKFI		Diff. in %
			Mean	SD	Mean	SD	
Gait event	$\textrm{t}_\textrm{SKF}$ (in %GC)	1.90e-21	39.00	0.26	43.67	0.37	11.98
	$\textrm{t}_\textrm{SAPF}$ (in %GC)	1.80e-21	46.25	0.30	48.77	0.18	5.45
	$\Delta \textrm{t}_\mathrm {SAPF-LLTD}$ (in %GC)	1.90e-21	1.62	0.33	-1.88	0.11	-215.55
TL imp.	$\Delta |{\textbf {p}}_\textrm{TL}|$ (in kg m/s)	1.97e-21	0.18	0.01	0.31	0.01	77.17
	$\Delta \textrm{p}_\textrm{TL,x}$ (in kg m/s)	1.97e-21	0.17	0.01	0.31	0.01	87.31
	$\Delta \textrm{p}_\textrm{TL,y}$ (in kg m/s)	1.97e-21	0.23	0.00	0.21	0.00	-9.81
RB imp.	$\Delta |{\textbf {p}}_\textrm{RB}|$ (in kg m/s)	1.60e-15	-0.16	0.02	-0.19	0.03	19.09
	$\Delta \textrm{p}_\textrm{RB,x}$ (in kg m/s)	2.07e-21	-0.04	0.03	-0.15	0.03	245.83
	$\Delta \textrm{p}_\textrm{RB,y}$ (in kg m/s)	1.97e-21	0.55	0.01	0.45	0.01	-19.64
CoM imp.	$\Delta |{\textbf {p}}_\textrm{CoM}|$ (in kg m/s)	4.74e-21	0.04	0.03	0.12	0.04	187.82
	$\Delta \textrm{p}_\textrm{CoM,x}$ (in kg m/s)	2.30e-13	0.12	0.03	0.16	0.04	32.74
	$\Delta \textrm{p}_\textrm{CoM,y}$ (in kg m/s)	1.97e-21	0.79	0.01	0.66	0.01	-16.74

The tested measures were: time of the start of knee flexion ($\textrm{t}_\textrm{SKF}$), time of the start of ankle plantar flexion ($\textrm{t}_\textrm{SAPF}$), the time period between the start of ankle plantar flexion and the leading leg touch-down ($\Delta \textrm{t}_\mathrm {SAPF-LLTD}$), the absolute ($\Delta |{\mathbf {p}}_\textrm{TL}|$), horizontal ($\Delta \textrm{p}_\textrm{TL,x}$), and vertical $(\Delta \textrm{p}_\textrm{TL,y})$ momentum change (impulse) of the trailing leg during the step-to-step transition, the the absolute ($\Delta |{\mathbf {p}}_\textrm{RB}|$), horizontal ($\Delta \textrm{p}_\textrm{RB,x}$), and vertical $(\Delta \textrm{p}_\textrm{RB,y})$ momentum change (impulse) of the remaining body during the step-to-step transition, and the the absolute ($\Delta |{\mathbf {p}}_\textrm{CoM}|$), horizontal ($\Delta \textrm{p}_\textrm{CoM,x}$), and vertical ($\Delta \textrm{p}_\textrm{CoM,y}$) momentum change (impulse) of the center of mass during the step-to-step transition. All tested differences were significant with a significance level of p < 0.001. The mean and standard deviation (SD) were calculated from 120 gait cycles, with the gait cycle starting at touch-down. The difference between the AKFI and PKFI experiments (Diff. in %) was calculated from the mean values as: $\frac{\textrm{mean}_\textrm{PKFI}- \textrm{mean}_\textrm{AKFI}}{\textrm{mean}_\textrm{AKFI}} \cdot 100$. x: horizontal direction, y: vertical direction, TL: Trailing Leg, RB: Remaining Body, CoM: Center of Mass of the whole robot, imp.: impulse = change inmomentum.

Between the start of the step-to-step transition and leading leg touch-down, all trailing leg and remaining body momentum changes are within 0.03kg m/s in horizontal direction and within 0.05kg m/s in vertical direction in the PKFI and in the AKFI experiments (Fig. 4, Supplementary Fig. S5 and S6). More than 50% of the above detailed changes happens between leading leg touch-down and the end of the step-to-step transition (Fig. 4, Supplementary Fig. S5 and S6).

The vertical and horizontal momentum changes of the CoM are significantly different between the AKFI and PKFI experiments Table 1. The momentum of the CoM increases by 0.16kg m/s and by 0.12kg m/s in the horizontal direction during the step-to-step transition with PKFI and with AKFI, respectively (Fig. 4e, Table 1, and Supplementary Fig. S5). The CoM’s vertical momentum increases by 0.13kg m/s more in the AKFI than in the PKFI experiment during the step-to-step transition. At the end of the step-to-step transition, the vertical momentum of the CoM reaches 0.31kg m/s and 0.29kg m/s with AKFI and with PKFI, respectively (Fig. 4f, Supplementary Table S3, and Supplementary Fig. S6).

The magnitude increase of the trailing leg momentum vector during the step-to-step transition is significantly larger (77%) in the PKFI experiment than in the AKFI experiment (p < 0.001) (Fig. 4, Table 1). The magnitude reduction of the remaining body momentum vector during the step-to-step transition is significantly larger (19%) in the PKFI experiment than in the AKFI experiment (p < 0.001) (Fig. 4, Table 1). The magnitude increase of the CoM momentum vector during the step-to-step transition is significantly larger (188%) in the PKFI experiment than in the AKFI experiment (p < 0.001) (Fig. 4, Table 1).

**Fig. 5:**
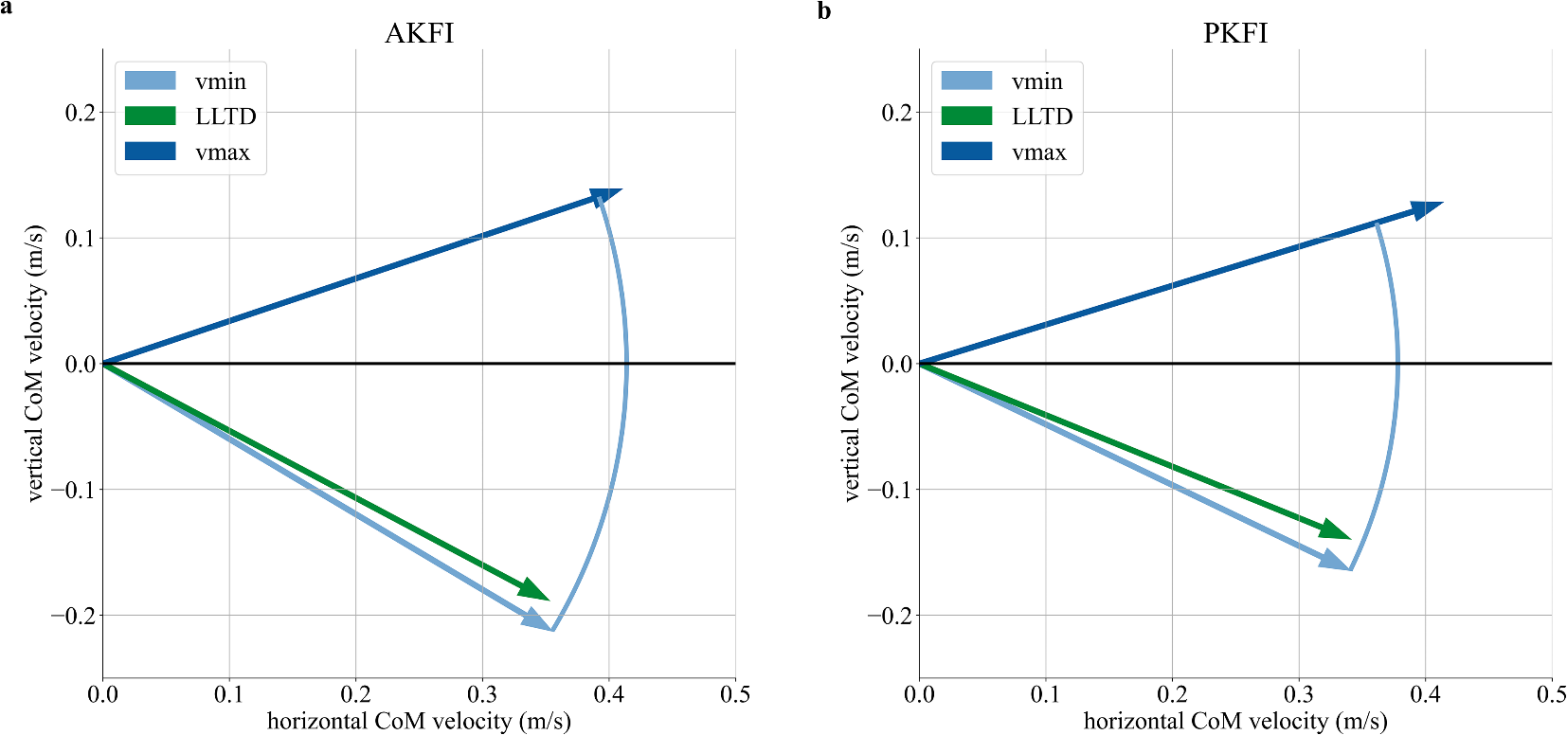
Center of Mass (CoM) velocity vectors at the start of the step-to-step transition (vmin), at leading leg touch-down (LLTD), and at the end of the step-to-step transition (vmax) in the active knee flexion initiation (AKFI - (**a**)) and in the passive knee flexion initiation (PKFI - (**b**)) experiments. An arc is drawn with a radius that is equal to the length of the velocity vector at vmin to better show the relation of the vector lengths at the three different times during the step-to-step transition. The length of the velocity vector increases between vmin and vmax more in the PKFI experiment than in the AKFI experiment.

The direction change of the CoM velocity vector during the step-to-step transition is 6.6deg larger in the AKFI experiment than in the PKFI experiment (Fig. 5, and Supplementary Fig. S7). The vertical component of the CoM velocity vector at the start of the step-to-step transition is 0.05m/s larger in the negative direction with AKFI than with PKFI (Fig. 5). At the end of the step-to-step transition, the horizontal components of the CoM velocity vectors are equal (0.41m/s), and the vertical components are 0.14m/s and 0.13m/s in the AKFI and in the PKFI experiment, respectively (Fig. 5, and Supplementary Table S4).

The center of mass total kinetic energy at the start of the step-to-step transition is 0.03J lower in the PKFI experiment than in the AKFI experiment. However, at the end of the step-to-step transition, the center of mass total kinetic energy reaches 0.21J in both the PKFI and the AKFI experiments (see Supplementary Fig. S8, and Supplementary Table S5).

The net positive cost of transport was 0.56 (SD: 0.05) in the AKFI experiment and 0.52 (SD: 0.03) in the PKFI experiment. A natural runner’s cost of transport with the same body mass m=2.1kg is $\text {COT}_\text {nr}={1.36}$^41,42^. Hence, the relative cost of transport ($\text {COT}_\text {re}$) of the EcoWalker-2 robot is 41% in the AKFI experiment, and 38% in the PKFI experiment, respectively.

## Discussion

The first goal of this study was to test Perry’s^5^ passive knee flexion process on a bipedal robot, as part of the lock-and-release mechanism of the human swing leg catapult. This investigation showed that the EcoWalker-2 robot is able to walk with sufficient toe clearance both with passive knee flexion initiation (PKFI) and with active knee flexion initiation (AKFI). The second aim of the study was to influence the ratio of energy flow into the remaining body and the trailing leg by indirectly manipulating the timing of the start of the ankle plantar flexion. This analysis showed that ankle plantar flexion starts significantly later in the PKFI experiment than in the AKFI experiment. This delay had a pronounced effect on the momentum changes during the step-to-step transition. Notably, the magnitude increase in the trailing leg momentum vector during the step-to-step transition was significantly larger (77%), the magnitude decrease in the remaining body momentum vector during the step-to-step transition was significantly larger (19%), and the magnitude increase in the center of mass momentum vector during the step-to-step transition was significantly larger (188%) in the PKFI experiment than in the AKFI experiment.

As expected, starting the knee flexion earlier in the AKFI experiment enables an earlier onset of ankle plantar flexion (Fig. 3). The timing delay of the start of ankle plantar flexion gives further evidence of Perry’s hypothesis that knee flexion is needed for ankle unloading^5^. Surprisingly, leading leg touch-down did not happen exactly at 50% of the gait cycle (GC) in either the PKFI or the AKFI experiment by neither legs (Fig. 3, see Supplementary Information S2). The 1-3%GC earlier or later leading leg touch-down could be a result of the slight asymmetry between the right and left sides of the robot. Even though the slack positions of the GAS and SOL spring-tendons were set symmetrically, there was always a little difference between the left and right, hip and knee joint actuators which causes a minor asymmetry in the robot gait. Asymmetry in human gait is also a common phenomenon, with strength imbalances present even in young adults. Research has shown that young adults typically exhibit strength asymmetries ranging from 5% to 15%^43,44^. In contrast, older adults tend to develop more pronounced asymmetries, typically in the range of 15% to 20%^43,45^.

The time period between the start of ankle plantar flexion and leading leg touch-down was expected to be shorter in the PKFI experiment than in the AKFI experiment. However, the delay in ankle plantar flexion onset was more pronounced than expected in the PKFI experiment. Specifically, ankle plantar flexion occurred 2%GC before leading leg touch-down in the AKFI experiment, similar to human data^40^, whereas it occurred 2%GC after leading leg touch-down in the PKFI experiment. The start of ankle plantar flexion means the start of ankle energy unloading where the ankle power curve becomes positive. Both in the PKFI and AKFI experiments, the event flow of the step-to-step transition can only start after the start of knee flexion. As the start of knee flexion is delayed in the PKFI experiment, all gait events of the trailing leg are delayed. In human experimental gait data, the start of knee flexion was reported to happen before the start of ankle plantar flexion and before leading leg touch-down^4,5^. Knee flexion appears to play a crucial role in initiating the step-to-step transition, with its timing having a cascading effect on subsequent gait events.

With the PKFI and AKFI experiments, two distinct disengagement processes were characterized on the same robotic system.

In the AKFI experiment, as ankle unloading started before leading leg touch-down, hip and knee flexion pulled the ankle joint upwards, making it possible for the ankle joint to start plantar flexing. Part of the unloading happened against the whole body weight before leading leg touch-down, a mechanism known as preemptive push-off, which has been well documented in human experiments^2,5,21,46,47^. In humans, preemptive push-off is initiated by gastrocnemius (GAS) and soleus (SOL) muscle activations, while in the robot, it is initiated by active hip and active knee flexion. After leading leg touch-down, only elastic recoil happens in humans as the observed GAS and SOL muscle activations diminish^2,5,21,46,47^. After leading leg touch-down, the robot’s hip and knee joints remain active, facilitating the rest of ankle unloading against the trailing leg through a dual mechanism of elastic recoil and active knee and hip flexion.

In literature, this preemptive push-off is explained as a method to restore heel-strike collision losses^11,27–29,48,49^. By using active knee and hip flexion with a passive ankle joint, a similar preemptive push-off could be reached. Notably, increasing hip work to facilitate leg swing has been shown to reduce ankle muscle activations during push-off^50^, which can be beneficial for reducing peak plantar pressures and treating conditions such as neuropathic plantar ulcers^51^. Conversely, studies have found that increasing ankle push-off work can decrease hip work^52–54^, highlighting the trade-offs between hip and ankle contributions during gait.

In the PKFI experiment, ankle unloading started only after leading leg touch-down, because hip flexion without active knee flexion initiation did not result in an upward pull at the ankle joint. As the ankle joint is passive, only elastic recoil from the GAS and SOL spring-tendons actuated the ankle plantar flexion. Unloading happened mainly against the trailing leg, as leading leg touch-down already happened.

Before leading leg touch-down, the trailing leg bears the whole body weight as the leading leg is in swing. It was hypothesized that the ankle energy being unloaded before leading leg touch-down mainly accelerates the remaining body. As the leading leg takes up increasingly more body weight after leading leg touch-down, the trailing leg becomes increasingly free to go into swing. The proportion of ankle energy unloaded after leading leg touch-down was expected to determine the distribution of energy between the trailing leg and the remaining body. A greater unloading of ankle energy after leading leg touch-down would result in a larger transfer of energy to the trailing leg, accelerating it into swing, while a smaller amount of energy would accelerate the remaining body. In the AKFI experiment, a small part of the ankle energy was already unloaded before leading leg touch-down but no part of the ankle energy was unloaded before leading leg touch-down in the PKFI experiment (see Supplementary Fig. S9). To check the hypothesis, the magnitude of the trailing leg and remaining body momentum vectors, and their horizontal and vertical components (Table 1, Fig. 4) at the start of the step-to-step transition (vmin), at leading leg touch-down, and at the end of the step-to-step transition (vmax) were analysed.

In line with the hypothesis, the trailing leg momentum vector’s magnitude increased significantly more in the PKFI experiment than in the AKFI experiment during the step-to-step transition (Table 1). The direction of the trailing leg momentum vectors at the start and end of the step-to-step transition reveals that the significant difference in magnitude increase is primarily due to the significant difference in the increase of the horizontal component of the trailing leg momentum. (Fig. 4). The trailing leg horizontal momentum started to increase at $\sim$ 20%GC in both the AKFI and PKFI experiments, but the increasing trend stopped when the knee motor torque command turned zero in the PKFI experiment (see Supplementary Fig. S5). The trailing leg horizontal momentum started to increase again only after the start of knee flexion in the PKFI experiment. Consequently, the trailing leg horizontal momentum reached a higher value until the start of the step-to-step transition in the AKFI experiment than in the PKFI experiment (see Supplementary Fig. S5). Nevertheless, the trailing leg horizontal momentum increased at a higher rate in the PKFI experiment than in the AKFI experiment, reaching a similar value at the end of the step-to-step transition. Similarly, human experimental walking data at 50-100% of the preferred transition speeds show a positive horizontal, vertical, and absolute trailing leg momentum change between the start of ankle plantar flexion and toe-off^4^. The similarity between this study’s experimental data and human walking data supports the idea that the observed trailing leg momentum dynamics are a key feature of efficient human gait.

Validating the hypothesis, the remaining body momentum increased less in the PKFI experiment than in the AKFI experiment. Actually, the magnitude decrease of the remaining body momentum vector during the step-to-step transition was significantly larger in the PKFI experiment than in the AKFI experiment (Table 1). The remaining body momentum vector change during the step-to-step transition was different between the PKFI and the AKFI experiment in both horizontal and vertical directions (Fig. 4). The horizontal component of the remaining body momentum vector decreased more during the step-to-step transition in the PKFI experiment than in the AKFI experiment, but it reached similar momentum values of 0.4kg m/s and 0.38kg m/s in the AKFI and in the PKFI experiment, respectively. The vertical component of the remaining body momentum vector increased more during the step-to-step transition in the AKFI experiment than in the PKFI experiment, but it reached the same 0.16kg m/s value at the end of the step-to-step transition. The remaining body horizontal momentum started to decrease at $\sim$ 35%GC (see Supplementary Fig. S5), while the remaining body vertical momentum started to decrease at $\sim$ 30%GC (see Supplementary Fig. S6) in both the AKFI and PKFI experiment, but the decrease rates were larger in the AKFI experiment than in the PKFI experiment. By the start of the step-to-step transition, the remaining body horizontal and vertical momentums reached a lower value in the AKFI than in the PKFI experiment (see Supplementary Fig. S5, see Supplementary Fig. S6). The larger rates of horizontal and vertical momentum decrease before the start of the step-to-step transition could be a consequence of the earlier start of knee flexion in the AKFI experiment. Similarly, human experimental walking data at 50-100% of the preferred transition speeds show a negative horizontal, and positive vertical and absolute remaining body momentum change between the start of ankle plantar flexion and toe-off^4^. These results suggest that this study’s experiments effectively capture the essential dynamics of human walking, particularly with respect to the remaining body momentum changes.

The trailing leg and remaining body horizontal momentums were complementing each other in both the AKFI and PKFI experiments, which makes the center of mass (CoM) horizontal momentum similar at vmin, leading leg touch-down, and vmax in the AKFI and the PKFI experiments (Fig. 4e, see Supplementary Fig. S5). As the trailing leg vertical momentum change was similar in the AKFI and PKFI experiments, the vertical momentum change of the remaining body is showing in the CoM vertical momentum changes (Fig. 4f, see Supplementary Fig. S6). The vertical momentum change difference comes from the vertical velocity component change of the CoM during the step-to-step transition (Fig. 5). As the CoM horizontal velocity components were similar in the AKFI and PKFI experiments, the change in the CoM vertical velocity components show as the change in the CoM velocity vector angles during the step-to-step transition (see Supplementary Fig. S7). Even though the CoM velocity vector angle change was larger in the AKFI experiment than in the PKFI experiment, the magnitude of the CoM velocity vector increased more in the PKFI experiment than in the AKFI experiment (Fig. 5). The larger magnitude increase of the CoM velocity vector in the PKFI experiment than in the AKFI experiment stems from the lower magnitude and smaller vertical component of the CoM velocity vector at vmin in the PKFI (Fig. 4f). The difference in the CoM vertical velocities at vmin can be explained by the remaining body’s vertical velocity differences (Fig. 4d), which could be a consequence of the earlier start of knee flexion in the AKFI experiment. The changes in the CoM velocity vector magnitudes show in the CoM total kinetic energy changes (see Supplementary Fig. S8). As a result of a larger negative remaining body vertical momentum at the start of the step-to-step transition, the total kinetic energy of the CoM was larger at the start of the step-to-step transition in the AKFI than in the PKFI experiment. As the remaining body vertical momentums were equal and the trailing leg vertical momentums were similar, the total kinetic energies of the CoM were equal at the end of the step to step transition in the AKFI and PKFI experiments.

While comparing robots with varying morphologies and numbers of legs has its limitations, it still offers valuable insights into how design of mechanics, actuators, and control influence energetic performance. Notably, the EcoWalker-2 robot’s best relative net cost of transport of 38%) is comparable to that of BiartHopper’s^55^, which achieved a relative net cost of transport of 45%. However, the latter robot features only a single hip actuator, whereas EcoWalker-2 robot utilizes two knee and two hip actuators for planar walking. With its design inspired by passive dynamic walkers, the Cornell biped^11^ achieved a relative cost of transport between 7% and 26% of a natural runner of similar weight. The calculation was based on the provided^11^ cost of transport range from 0.05 (mechanical) to 0.2 (total, electrical) at 13kg body weight. Therefore, the EcoWalker-2 robot approaches the efficiency of passive-dynamic walkers, while remaining fully controllable and versatile. Three causes for the EcoWalker-2 robot’s outstanding energetic efficiency can be identified. First, its brushless motors, low gear ratio, and efficiently built actuators contribute to the robot’s low cost of transport^56^. Second, the EcoWalker-2 robot walks with straight legs in mid-stance. Without an acting moment arm, the robot’s knee actuator is effectively unloaded around midstance. Third, the EcoWalker-2 robot’s leg design and control allows harnessing the elastic energy in the spring-loaded ankles. The robot’s catapult mechanism then converts the stored energy into combined swing leg motion and forward momentum. So far, the EcoWalker-2 robot is freely walking in the sagittal plane, but it is constrained against lateral motions and pitch rotation. Once a 3D-walking robot, the EcoWalker-2 robot’s cost of transport is expected to increase due to the required, additional actuation.

This study has limitations as the experiments were done on a bipedal robot which can only walk in sagittal plane and its upper body is stabilized by a 4-bar mechanism. The restrictions of the robot’s design and experimental setup could have potentially over emphasized the results as the upper body stabilization and 3D stabilization of the robot were not factored in. Focusing on the robot’s leg mechanics enabled a more focused evaluation of sagittal plane effects with minimal confounding factors.

Human gait data is highly variable, especially if it is about patient population^24^, whereas gait events in robots are more reliable, SD < 0.4%GC (see Supplementary Table S2, and Supplementary Table S6. The time sequence of gait events around the step-to-step transition (leading leg touch-down, start of ankle plantarflexion, start of knee and hip flexion) from human gait data is not defined clearly in literature^5,57,58^. A robotic system makes the gait event identification more reliable, which in turn makes gait analysis, based on gait events, and analysis of event flows more accurate. On a robotic system, the push-off event flow, momentum and energy flow can be investigated more reliably than with human measurements. However the transfer of the results to human gait remains to be explored in future studies. To take the natural differences of human gait into account, future robot experiments could vary mechanical and control parameters, including the purposeful insertion of left-right asymmetries, altering segment masses, or varying actuator gains that simulate lower muscle power. Additionally, future studies could focus on patients with prosthetic or orthotic devices that enable passive knee flexion initiation.

Changing the way of knee flexion initiation changes the timing of the gait events, remaining body, trailing leg and CoM velocities, momentums, and kinetic energies during the step-to-step transition. Although the method of knee flexion initiation has a significant impact during the step-to-step transition, its effect diminishes thereafter and remains negligible for the first $\sim$ 20% of the gait cycle. Moreover, in terms of walking speed and cost of transport, the way of knee flexion initiation does not have a large overall effect on the whole gait cycle which also shows the robustness of human-like walking gaits. Changes in the gait during the step-to-step transition change the interplay between the disengagement process of the trailing leg and the collision process of the leading leg with the ground.

By manipulating the way of knee flexion initiation, the timing of the gait events around push-off, the distribution of kinetic energy, showing in velocity and momentums of the trailing leg and remaining body, the redirection of the CoM velocity vector and the CoM kinetic energy can be influenced. Knee flexion initiation has been identified as a critical factor in ankle unloading and the coordination of gait events at the end of stance, providing insights into the biomechanical mechanisms underlying the step-to-step transition in human walking.

These findings have implications for the development of humanoid robots, for the development of assistive technologies, including prosthetics, and orthotics, and may inform gait rehabilitation practices that target the critical step-to-step transition phase. Specifically, enabling passive knee flexion initiation could be beneficial in humanoid robots with passive ankles, in ankle-knee orthoses with passive ankles, and in ankle-knee prostheses with passive ankles for saving on control effort, and reducing hardware complexity otherwise required for active knee flexion before the step-to-step transition. Furthermore, humanoid robots and assistive devices with passive ankles could benefit from a delayed or passive knee flexion onset to gain more horizontal trailing leg momentum during the step-to-step transition. This study also informs gait rehabilitation practices about the importance of knee flexion timing as knee flexion enables hip flexion and ankleplantarflexion. Patients with problems initiating hip flexion or ankle plantarflexion during the step-to-step transition may have the origin of their problems with knee flexion initiation. Addressing these deficits through knee flexion initiation assistance could be an option for these patients.

## Methods

**Fig. 6:**
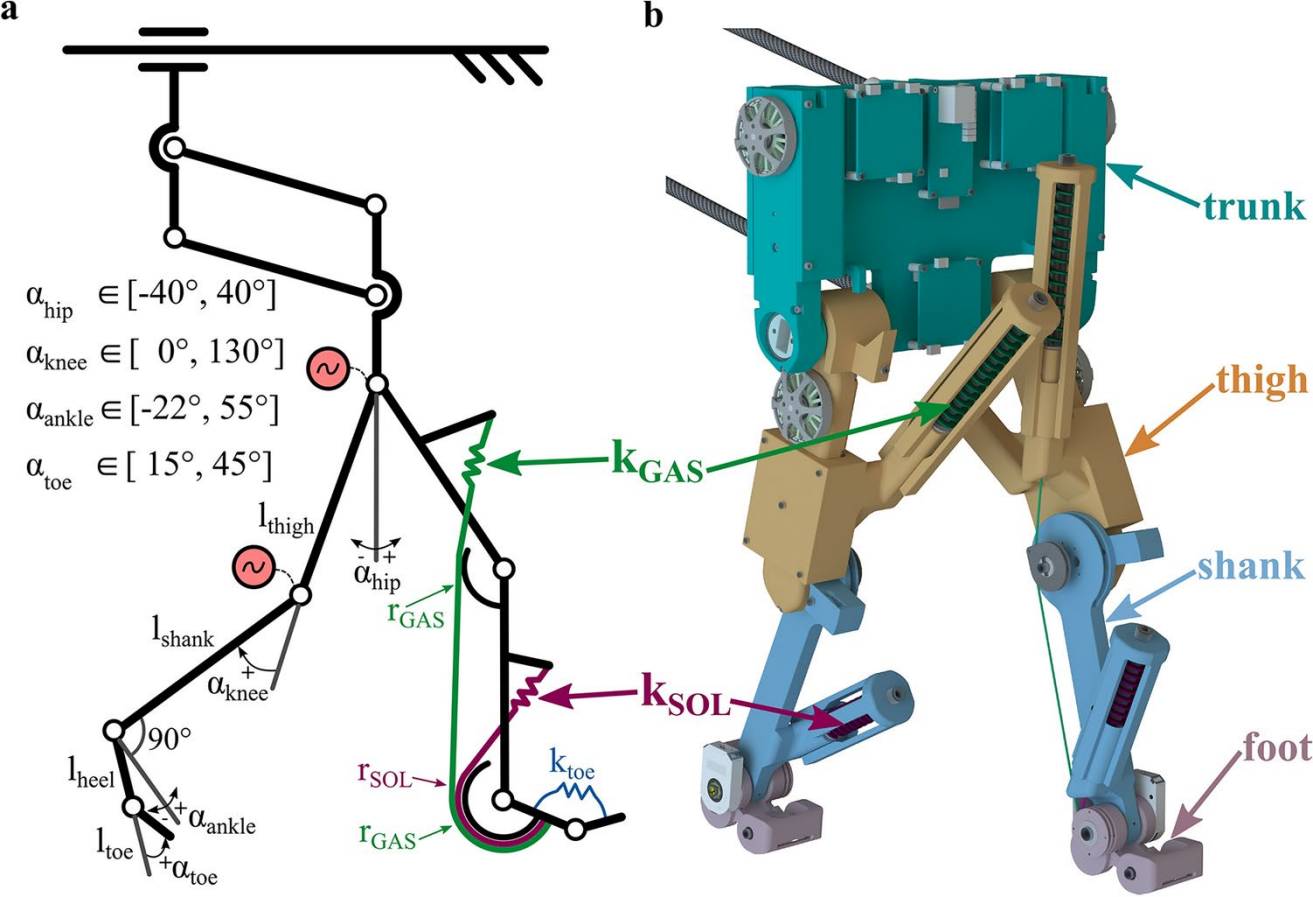
Schematic and rendering of the bipedal EcoWalker-2 robot. **a:** Schematic of the robot with spring-tendon routing, angle definitions, and range of motion of the joints. Segment lengths: $\mathrm{l}_\mathrm{thigh}=\mathrm{l}_\mathrm{shank}={160}\mathrm{mm}$, $\mathrm{l}_\mathrm{heel}={32}\mathrm{mm}$, $\mathrm{l}_\mathrm{toe}={17}\mathrm{mm}$. Pulley radii: $\mathrm{r}_\mathrm{GAS}=\mathrm{r}_\mathrm{SOL}={13}\mathrm{mm}$. Spring stiffness values: $\mathrm{k}_\mathrm{GAS}={1.4}\mathrm{N/mm}$, $\mathrm{k}_\mathrm{SOL}={4.5}\mathrm{N/mm}$, $\mathrm{k}_\mathrm{toe}={8.04}\mathrm{Nmm/deg}$. The robot’s trunk movement is constrained to translations in the sagittal plane, and all trunk rotations are locked. The four-bar guide slides freely in fore-aft direction. **b:** Rendering of the bipedal robot with segment name indications.

The EcoWalker-2 robot, a bipedal, human-like robot with hip and knee motors, and spring-loaded passive ankles, was used in the experiments. Building upon the original EcoWalker robot^12^, the updated EcoWalker-2 received an update to its design freeing up the movement of the knee joint while preserving toe clearance during swing phase. The robot’s control was updated to switch the knee actuation passive during specified times of the gait cycle (GC). A calibration step was also introduced prior to each experiment to symmetrically set the slack length of the ankle spring-tendons. Experiments with the EcoWalker-2 robot with active knee joint actuation during the whole gait cycle (AKFI) and with passive knee joint actuation in more than 50% GC (PKFI) were conducted. To ensure comparability, the hip control, ankle spring-tendon properties, and slack lengths were kept identical in both experiments.

### Update of the bipedal robot design

To enable passive knee flexion in the EcoWalker-2 robot, the passive knee extending structures of the original EcoWalker robot^12^ were removed. Specifically, the toe tendon, which pulled up the toes for ground clearance when the knee was flexed, and the VAS spring-tendon, representing the vasti muscle-tendon unit, were removed. In the EcoWalker-2 robot, only the GAS spring-tendon, representing the gastrocnemius muscle-tendon unit, and a SOLO actuator module^56^ act at the knee joint. The SOLO actuator modules are driven by brushless DC (BLDC) motors. To maintain toe clearance during swing, the robot’s heel segment was shortened from 48mm to 32mm and the robot’s toe segment was shortened from 24mm to 17mm (Fig. 6). To dampen the touch-down of the heel, a 2mm thick silicone layer (shore hardness 00-50) was glued to the bottom of the heel segment. The ankle joints are passive, spring-loaded by the SOL and GAS spring-tendons, representing the soleus and gastrocnemius muscle tendon units. Prior to each experiment, a calibration step was introduced to set the slack length of the SOL and GAS ankle spring-tendons symmetrically and accurately. To maintain constant slack lengths of the spring-tendons throughout the experiments, two measures were employed. Firstly, anti-vibration glue (Vibra-Tite VC-3 Threadmate) was applied to the setscrews used for adjusting the slack lengths, thereby securing them in place. Secondly, a spring cage was installed around the SOL and GAS spring mechanisms to prevent any unwanted rotation.

In the EcoWalker-2 robot, no changes were made to the hip, and toe joint actuators of the original EcoWalker robot^12^. The hips are actuated by SOLO actuator modules, and rotational toe springs act around the toe joints, representing the foot compliance (Fig. 6).

The stiffnesses of the used springs are the following: SOL spring: 4.5N/mm, GAS spring: 1.4N/mm, toe spring: 8.04Nmm/deg. The SOL and GAS spring-tendons are acting on the ankle at a constant moment arm of 13mm, and the GAS spring-tendon is acting on the knee joint at a constant moment arm of 13mm. The slack length of both the SOL and GAS spring-tendons were set to 0mm at -22$^{\circ }$ ankle joint and 0$^{\circ }$ knee joint angles for both legs in all experiments. The rotational toe springs are loaded in stance from an initial toe angle of 15$^{\circ }$ where the toe spring torque is zero. Nomenclature and the zero-angle definitions are shown in Fig. 6.

### Update of the robot control

Central Pattern Generators (CPGs), amplitude controlled phase oscillators, were implemented for both hip and knee joints with a half-cycle phase shift between left and right oscillator nodes^12,59^. Reference trajectories for both joints were extracted from a 2D neuromuscular simulation of human walking^60^ to design the commanded joint trajectories in the CPG control. The same CPG control parameters were used in all experiments (Table 2) to make them comparable. The frequency (*f*) defines the length of one gait cycle, flexion factors ($F_\mathrm{hip}$ and $F_\mathrm{knee}$) define the start of hip and knee flexion separately, as a fraction of the gait cycle. The knee amplitude $(\Theta _\mathrm{kneeAmplitude})$ and offset ($\Theta _\mathrm{kneeOffset}$) parameters define the knee trajectory. The hip amplitude ($\Theta _\mathrm{hipAmplitude}$) and offset ($\Theta _\mathrm{hipOffset}$) parameters define the hip trajectory. The hip swing steady parameter ($\varphi _\mathrm{hip}$) defines the hip’s steady duration at the end of the swing, as a fraction of a gait cycle.

**Table 2:** CPG control parameters for all experiments.

Parameter		Value
Frequency	*f*	1Hz
Hip duty factor	$F_\mathrm{hip}$	0.6%GC
Knee duty factor	$F_\mathrm{knee}$	0.6%GC
Knee amplitude	$\Theta _\mathrm{kneeAmplitude}$	55$^{\circ }$
Knee offset	$\Theta _\mathrm{kneeOffset}$	8$^{\circ }$
Hip amplitude	$\Theta _\mathrm{hipAmplitude}$	26$^{\circ }$
Hip offset	$\Theta _\mathrm{hipOffset}$	12$^{\circ }$
Hip swing steady	$\varphi _\mathrm{hip}$	0.05%GC

GC: gait cycle.

The CPG-generated hip and knee reference trajectories were the input of a PD controller which calculated the commanded hip and knee motor currents. Due to slight mechanical differences, the hip actuators of the right and left leg performed mildly different. Hence, P gains of the hip motors were manually tuned to obtain matching left and right leg kinematics: $k_{p,\mathrm{hip, right}} = 30$, $k_{p,\mathrm{hip, left}} = 26$. The D gain of the hips, and the PD gains of the knees were set symmetrically: $k_{d,\mathrm {hip, right/left}} = 0.2$, $k_{p,\mathrm {knee, right/left}} = 7$, $k_{d,\mathrm {knee, right/left}} = 0.2$.

In the active knee flexion initiation (AKFI) control set the knee motor was controlled to follow the CPG-defined knee angle curve in the whole gait cycle. For the passive knee flexion initiation (PKFI) control set the knee motor’s control was modified to send zero torque command to the motor in the specified time window during a gait cycle (see Supplementary Fig. S2). The PKFI mode was activated through a keyboard input, once the robot was in steady-state locomotion, typically after 2 gait cycles.

### Experimental setup and data acquisition

Two knee motor modes were compared: PKFI and AKFI. In the PKFI experiment, the start of the zero knee motor torque period was set to 35% GC (see Supplementary Fig. S2). The start of the period was chosen in the middle of the stance phase of the robot, so that the start time is after the loading response but before the start of push-off. The start of push-off was defined as the start of ankle plantar flexion. The knee motor torque remained zero until the end of the same gait cycle (see Supplementary Fig. S2). One gait cycle starts at the touch-down of the foot and lasts until the next touch-down of the same foot.

To ensure the independence of the results from the exact start time of the zero knee motor torque period, an additional experiment was conducted where the zero torque was applied to the knee motor at 40% GC, instead of 35% GC (PKFI40 experiment). The data analysis of the PKFI40 experiment is available in the Supplementary Information S3.

In the PKFI experiment, knee flexion onset emerged dynamically. PKFI knee flexion onset was observed at 47% GC (Fig. 3). The CPG-controlled active knee flexion initiation in the AKFI experiment was set prior to 47% GC, at 40% GC. Due to a $\sim$ 20% GC shift between the observed touch-down of the foot and the start of the CPG defined gait cycle, 40% GC translates into $F_\mathrm{knee}=0.6$ knee flexion factor in the CPG parameters (Table 2).

Hip motor control and the CPG parameters (Table 2) were not changed between the PKFI and AKFI experiments. The robot’s trunk was constrained to translations in the sagittal plane, and all trunk rotations were locked with a four-bar mechanism in all experiments.

The setup was instrumented to measure the kinematics of the robot with rotary encoders at the hip and knee joints (AEDT-9810, Broadcom, 5000 CPR), at the four-bar mechanism, at the ankle joint (AMT-102, CUI, 4096 CPR), and on the treadmill (AEAT8800, Broadcom, 4096 CPR). The robot’s forward motion was measured with two linear potentiometers on the four-bar slider (LX-PA-50, WayCon). The power consumption of the right and left hip and knee motors were measured individually by current sensors (ACHS-7121, Broadcom) connected to each motor’s separate motor driver boards. All sensors were sampled around 600Hz by a single board computer (4B, Raspberry Pi Foundation).

To avoid instabilities caused by a fixed control loop length, a dynamic sampling approach was adopted. Instead of sampling at a constant frequency, the sampling was synchronized with the completion of each control loop cycle, sending the next command only after the previous cycle had finished executing.

### Data analysis

The collected data was trimmed to 120s (120 gait cycles) during which the robot was steady-state walking. Since sampling intervals were not constant, sensor data were interpolated to 1000Hz sampling rate before analysis. Touch-down, the start of a gait cycle, was defined as the moment where the ankle joint angle starts to change after its approximately constant value ($\sim$ 22deg plantarflexion) during swing. An averaged gait cycle data was calculated from each measured sensor data, starting from touch-down. Butterworth digital low-pass zero phase shift filters with second order sections output type were applied^61^. Separate Butterworth filter orders and cut-off frequencies were set depending on the data/sensor type, which are given in the Supplementary Table S1. In all result plots, the averaged left leg data starting with the left foot touch-down is presented. Equivalent plots for the averaged right leg data in the gait cycles starting with the right foot touch-down is available in the Supplementary Information S2.

The ankle joint power (${\mathcal {P}}_\mathrm{A}$), and the GAS spring-tendon’s contribution to the knee joint power (${\mathcal {P}}_\mathrm{K, GAS}$) was calculated as:

1 $$\begin{aligned}\mathcal{P}_\mathrm{A} = \begin{cases}\begin{array}{l}\omega_\mathrm{A} \cdot (\mathrm{k}_\mathrm{SOL} \cdot \mathrm{r}_\mathrm{SOL} \cdot (\alpha_\mathrm{A} \cdot \mathrm{r}_\mathrm{SOL} - \mathrm{l}_\mathrm{SOL, slack}) +... \\  \mathrm{k}_\mathrm{GAS} \cdot \mathrm{r}_\mathrm{GAS} \cdot ((\alpha_\mathrm{A} - \alpha_\mathrm{K}) \cdot \mathrm{r}_\mathrm{GAS} - \mathrm{l}_\mathrm{GAS, slack})),\vspace{0.5cm}\end{array} & \text{if} \quad \alpha_\mathrm{A} \geq \alpha_\mathrm{K}\\\begin{array}{l}\omega_\mathrm{A} \cdot \mathrm{k}_\mathrm{SOL} \cdot \mathrm{r}_\mathrm{SOL} \cdot (\alpha_\mathrm{A} \cdot \mathrm{r}_\mathrm{SOL} - \mathrm{l}_\mathrm{SOL, slack}),\end{array} & \text{else}\end{cases}\end{aligned}$$

2 $$\begin{aligned}    \mathcal{P}_\mathrm{K, GAS} = \omega_\mathrm{K} \cdot \mathrm{k}_\mathrm{GAS} \cdot \mathrm{r}_\mathrm{GAS} \cdot ((\alpha_\mathrm{A} - \alpha_\mathrm{K}) \cdot \mathrm{r}_\mathrm{GAS} - \mathrm{l}_\mathrm{GAS, slack}),\end{aligned}$$

where $\omega _\mathrm{A}$ is the angular velocity of the ankle in rad/s, $\omega _\mathrm{K}$ is the angular velocity of the knee in rad/s, $\alpha _\mathrm{A}$ and $\alpha _\mathrm{K}$ are angle values of the ankle and knee joints in radian, $\textrm{k}_\mathrm{SOL}$ and $\textrm{k}_\mathrm{GAS}$ are spring stiffness values of SOL and GAS respectively in N/m, $\textrm{r}_\mathrm{SOL}$ and $\textrm{r}_\mathrm{GAS}$ are pulley radii of SOL and GAS respectively in meter, $\textrm{l}_\mathrm{SOL, slack}$ and $\textrm{l}_\mathrm{GAS, slack}$ are the slack lengths of SOL and GAS spring-tendons respectively in meter.

The step-to-step transition was analyzed to see the effects of both the push-off of the trailing leg and the touch-down of the leading leg. Step-to-step transition was defined from minimum to maximum vertical center of mass (CoM) velocity^17^. In the velocity plots, known as ’CoM hodographs’^17^, from this study’s experiments (see Supplementary Fig. S1), two similar height vertical maximum peaks following the minimum CoM vertical velocity can be consistently observed. The second CoM vertical velocity peak was selected for analysis, as it marks the point where the velocity vector is redirected not only vertically but also horizontally (see Supplementary Fig. S1), indicating the completion of the transition^17^.

The following gait events were identified during post-processing: Start of Ankle Plantar Flexion (SAPF), Toe-Off (TO), Leading Leg Touch-Down (LLTD), Start of Hip Flexion (SHF), and Start of Knee Flexion (SKF) (Fig. 2).

Start of Ankle Plantar Flexion (SAPF) occurs when the trailing leg’s ankle starts to plantarflex after steadily dorsiflexing during stance. The moment of SAPF was defined when the trailing leg’s ankle plantar flexion angle reached its maximum.

Toe-Off (TO) of the trailing leg occurs when the trailing leg’s foot leaves the ground. After TO, the ankle angle is approximately constant in swing (Fig. 2); at the time of TO, the ankle dorsiflexion angle reaches its minimum and then stops to change. The gradient^62^ of the ankle angle was calculated, and defined the moment of TO when the smallest ankle dorsiflexion angle occurred within a 40ms time window after the largest negative peak in the ankle angle gradient.

Leading Leg Touch-Down (LLTD) is identified when the foot of the leading leg makes contact with the ground. Since the ankle angle remains relatively constant during the swing phase, LLTD can be detected by monitoring changes in the leading leg ankle angle. The gradient^62^ of the leading leg ankle angle was calculated, and defined LLTD as the last instance before TO when the absolute value of the instantaneous ankle angle gradient exceeded 1rad/s, indicating the onset of plantarflexion or dorsiflexion.

Start of Hip Flexion (SHF) occurs when the hip starts flexing after steadily extending during stance. The gradient^62^ of the hip angle was calculated, and defined the moment of SHF when the value of the instantaneous hip angle gradient was more than 1rad/s. The time period of interest for the search started from the moment of peak hip extension angle and lasted until the end of the gait cycle.

Start of Knee Flexion (SKF) occurs when the knee starts to flex during stance phase. The gradient^62^ of the knee angle was calculated, and defined the moment of SKF when the value of the instantaneous knee angle gradient was more than 1rad/s. The time period of interest for the search started from 0.2s before SHF and lasted until 0.1s after SHF.

The robot was considered as a system of segments, divided into two feet, two shanks (lower leg), two thighs (upper leg), and a trunk (Fig. 6b). The velocities at each segment’s center of mass were calculated using the joint position angles from the encoders at the ankle, knee, and hip joints, at the four-bar mechanism’s lower rod, and the potentiometer values that measured the horizontal position of the four-bar slider connector. Using the segments’ center of mass velocity vectors (${\mathbf{v}}_\textrm{i}$), and the segments’ masses ($m_\textrm{i}$), the instantaneous linear momentum vectors of each segment (${\mathbf{p}}_\textrm{i}$) were calculated:

3 $$\begin{aligned} {\mathbf{p}}_\textrm{i} = m \cdot {\mathbf{v}}_\textrm{i}, \end{aligned}$$

where $\mathrm{i}$ indicates one of the body segments: foot, shank, or thigh of the trailing or leading leg, or the trunk.

From the momentum vectors of the segments, the following measures were calculated: momentum and velocity vectors, and linear kinetic energies of the trailing leg, remaining body, and the CoM (see Supplementary Information S1). The active joint power of the knee and hip joints could be calculated separately for each joint, as each motor was connected to a separate motor driver board and current sensor:

4 $$\begin{aligned} P_\mathrm{joint, side} = I_\mathrm{cal, joint, side} \cdot U_\mathrm{supply}, \end{aligned}$$

where $P_\mathrm{joint, side}$ is the active knee/hip joint power on the trailing/leading leg side of the robot, $I_\mathrm{cal, joint, side}$ is the measured net current, at the motor driver board of the knee/hip joint on the trailing/leading leg side of the robot, and $U_\mathrm{supply}$ is the supply voltage of the robot, 24V. For one gait cycle, the net positive energy consumption of the two knee and two hip motors was calculated as the average of the sum of the knee and hip motor powers, after subtracting the idle power consumption of the motor drivers, and removing the negative power values. The robot’s net positive cost of transport ($\mathrm{COT}$) was calculated as $\mathrm{COT}=\mathrm{E}_\mathrm{en}/(mgv)$, with $\mathrm{E}_\mathrm{en}$ as the net positive energy consumption of the knee and hip motors^63,64^, *m* as the robot’s mass, *g* as the gravitational acceleration (9.81${\hbox {m/s}}^{2}$), and *v* as the average horizontal robot speed over 120 gait cycles. Comparing the EcoWalker-2 robot’s net positive cost of transport ($\text {COT}_\text{en}$) in a fair manner to that of other legged hoppers, bipedal robots, and quadrupedal robots of different body mass can be achieved by calculating the robot’s relative cost of transport ($\text {COT}_\text {re}$)^42^. Hence, the ratio between the robot’s net positive cost of transport and the cost of transport of a natural runner ($\text {COT}_\text {nr}$) of equal body mass $\text {COT}_\text {re}=\text {COT}_\text {en}/\text {COT}_\text {nr}\cdot {100}{\%}$^41^ were calculated.

Wilcoxon signed-rank test^61^ was used to test the differences between the AKFI and the PKFI experiments for significance. In each experiment, data was collected from 120 gait cycles, providing a sample size of $\textrm{N} = 120$ for the statistical significance tests. Nonparametric, paired-samples Wilcoxon test was used because the samples did not show normal distribution, and the samples were measured on the same robotic system, so the measurements are dependent^65^. The following measures were tested: the time of the start of knee flexion in the AKFI and PKFI experiments, time of the start of ankle plantar flexion in the AKFI and PKFI experiments, the time period between start of ankle plantar flexion and leading leg touch-down in the AKFI and PKFI experiments, the absolute, horizontal, and vertical momentum change (impulse) of the trailing leg during the step-to-step transition in the AKFI and PKFI experiments, the absolute, horizontal, and vertical momentum change (impulse) of the remaining body during the step-to-step transition in the AKFI and PKFI experiments, and the absolute, horizontal, and vertical momentum change (impulse) of the CoM during the step-to-step transition in the AKFI and PKFI experiments.

## Supplementary Information

Supplementary Information 1.

Supplementary Information 2.

Supplementary Information 3.

## Data Availability

All data needed to evaluate the conclusions of the paper are available in the paper or the Supplementary Materials. CAD design files of the EcoWalker-2 robot, control code, data analysis and visualization code, and experimental data are available for non-commercial use at https://doi.org/10.17617/3.BJ584M. Video footage of the EcoWalker-2 robot’s locomotion on the treadmill in both active knee flexion initiation (AKFI) and passive knee flexion initiation (PKFI) experiments is available at https://www.youtube.com/watch?v=RupuZPBI6Bg and at https://www.youtube.com/watch?v=oWwJbTPUOM4.
