# Supplementary material for: Passive knee flexion increases forward impulse of the trailing leg during the step-to-step transition
Source: Sci Rep. 2025 Apr 15;15:12915. doi: 10.1038/s41598-025-95589-4 (PMC12000429; doi:10.1038/s41598-025-95589-4)
Supplement: Supplementary file 1 — Supplementary Information 1. [file 41598_2025_95589_MOESM1_ESM.pdf]

Supplementary Materials for

Passive knee flexion increases forward impulse of the trailing leg during the step-to-step transition

Bernadett Kiss[1,*], Alexandra Buchmann[2], Daniel Renjewski[2], and Alexander Badri-Spröwitz[1,3]

[1]Max Planck Institute for Intelligent Systems, Stuttgart, 70569, Germany
[2]Technical University of Munich, Chair of Applied Mechanics, TUM School of Engineering & Design, Department of Mechanical Engineering, Garching near Munich, 85748, Germany
[3]KU Leuven, Department of Mechanical Engineering, Leuven, 3000, Belgium
[*]kiss@is.mpg.de

ABSTRACT

The PDF file includes:

Supplementary Information S1 to S3
Figure S1 to S21
Tables S1 to S9
Captions for Movies S1 and S2

Other Supplementary Material for this manuscript includes the following:

Movie S1: https://www.youtube.com/watch?v=RupuZPBI6Bg
Movie S2: https://www.youtube.com/watch?v=oWwJbTPUOM4

CAD design files of the EcoWalker-2 robot, control code, data analysis and visualization code, and experimental data: https://doi.org/10.17617/3.BJ584M

1 Supplementary Information S1. Momentum, velocity vector angle, and linear kinetic energy calculation method

From the momentum vectors of the segments, we calculated the momentum vector of the trailing leg ($\mathbf{p}_{TL}$), and the momentum vector of the remaining body ($\mathbf{p}_{RB}$):

$$\mathbf{p}_{TL} = \mathbf{p}_{TL,foot} + \mathbf{p}_{TL,shank} + \mathbf{p}_{TL,thigh}, \tag{1}$$

$$\mathbf{p}_{RB} = \mathbf{p}_{LL,foot} + \mathbf{p}_{LL,shank} + \mathbf{p}_{LL,thigh} + \mathbf{p}_{trunk}, \tag{2}$$

where $\mathbf{p}_{TL,foot}$ is the momentum vector of the trailing leg's foot segment, $\mathbf{p}_{TL,shank}$ is the momentum vector of the trailing leg's shank segment, $\mathbf{p}_{TL,thigh}$ is the momentum vector of the trailing leg's thigh segment, $\mathbf{p}_{LL,foot}$ is the momentum vector of the leading leg's foot segment, $\mathbf{p}_{LL,shank}$ is the momentum vector of the leading leg's shank segment, $\mathbf{p}_{LL,thigh}$ is the momentum vector of the leading leg's thigh segment, $\mathbf{p}_{trunk}$ is the momentum vector of the trunk segment.
From the trailing leg's and the remaining body's momentum vectors, the trailing leg's and remaining body's velocity vectors at their respective center of masses could be calculated (Equations (3) and (4)). Using the trailing leg's and remaining body's momentum vectors, the momentum vector and velocity of the whole robot's center of mass (CoM) could be calculated (Equation (5)).

$$\mathbf{p}_{TL} = m_{TL} \cdot \mathbf{v}_{TL}, \tag{3}$$

$$\mathbf{p}_{RB} = m_{RB} \cdot \mathbf{v}_{RB}, \tag{4}$$

$$\mathbf{p}_{CoM} = m_{CoM} \cdot \mathbf{v}_{CoM} = \mathbf{p}_{TL} + \mathbf{p}_{RB}, \tag{5}$$

where $\mathbf{p}_{TL}$ is the linear momentum vector of the trailing leg at the CoM of the trailing leg, $\mathbf{p}_{RB}$ is the linear momentum vector of the remaining body at the CoM of the remaining body, $\mathbf{p}_{CoM}$ is the linear momentum vector of the whole body at the whole body CoM, m_{TL} is the mass of the trailing leg, m_{RB} is the mass of the remaining body, m_{CoM} is the mass of the whole body, $\mathbf{v}_{TL}$ is the linear velocity vector of the trailing leg at the CoM of the trailing leg, $\mathbf{v}_{RB}$ is the linear velocity vector of the remaining body leg at the CoM of the remaining body, $\mathbf{v}_{CoM}$ is the linear velocity vector of the whole body at the CoM of the whole body.
The instantaneous CoM velocity vector angle (α) could be calculated as the angle between the horizontal direction and the CoM velocity vector (Equation (6)). Positive α angle means a counter-clockwise turn from the horizontal direction.

$$\alpha = \arctan(\frac{v_{CoM,y}}{v_{CoM,x}}), \tag{6}$$

where $v_{CoM,y}$ is the vertical, and $v_{CoM,x}$ is the horizontal component of the CoM velocity vector ($\mathbf{v}_{CoM}$).
Knowing all segments', the trailing leg's, the remaining body's, and the CoM's velocity vectors and masses, we can calculate the instantaneous linear kinetic energies of each of these segments/segment groups in both horizontal and vertical directions:

$$E_{k,x,i} = \frac{1}{2} \cdot m_i \cdot v_{x,i}^2, \tag{7}$$

$$E_{k,y,i} = \frac{1}{2} \cdot m_i \cdot v_{y,i}^2, \tag{8}$$

where $E_{k,x,i}$ and $E_{k,y,i}$ are the horizontal and vertical components of the kinetic energies of segment/segment group i, m_i is the mass of segment/segment group i, $v_{x,i}$ and $v_{y,i}$ are the horizontal and vertical velocity vector components of segment/segment group i.

2 Center of mass hodographs

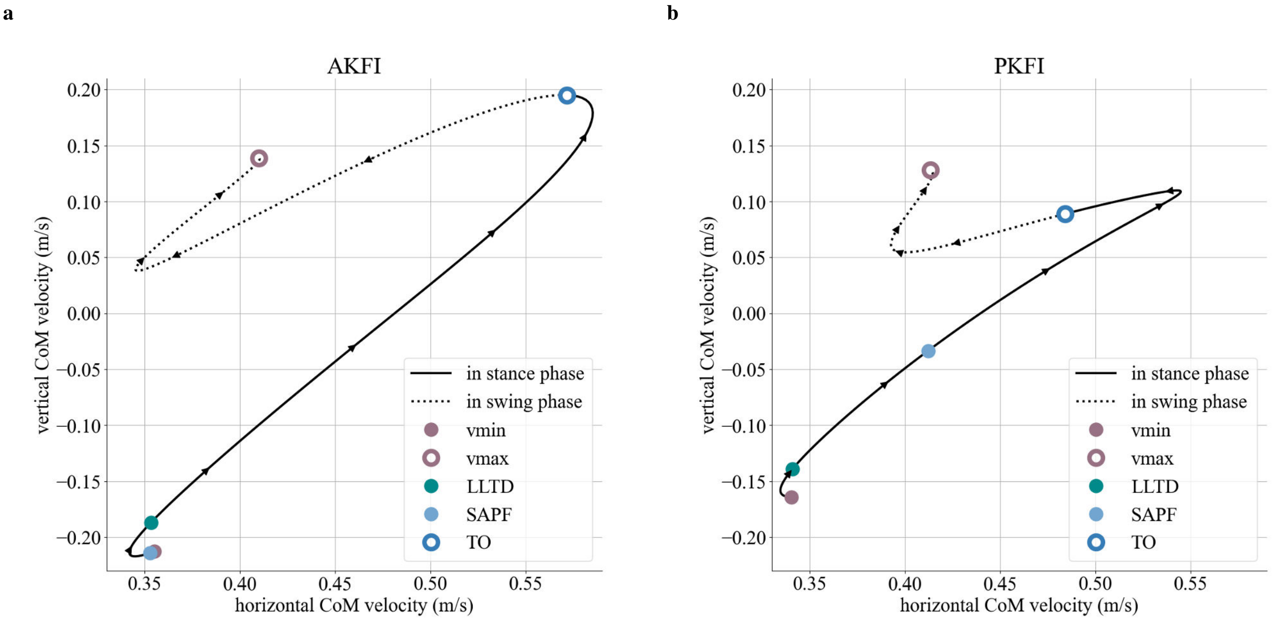

Figure S1. Center of Mass (CoM) instantaneous velocity vector plot (CoM hodograph[1]). The velocity vector's origin is placed in $(0,0)$, the end of the velocity vector draws the hodograph during the step-to-step transition period. The filled circle at 'vmin' and unfilled circle at 'vmax' show the beginning and the end of the step-to-step transition period.
Abbreviations: vmin: time of minimum vertical velocity of the CoM, vmax: second vertical velocity peak of the CoM after vmin, LLTD: Leading Leg Touch-Down, SAPF: Start of Ankle Plantar Flexion, TO: Toe-Off.

3 Current command plot

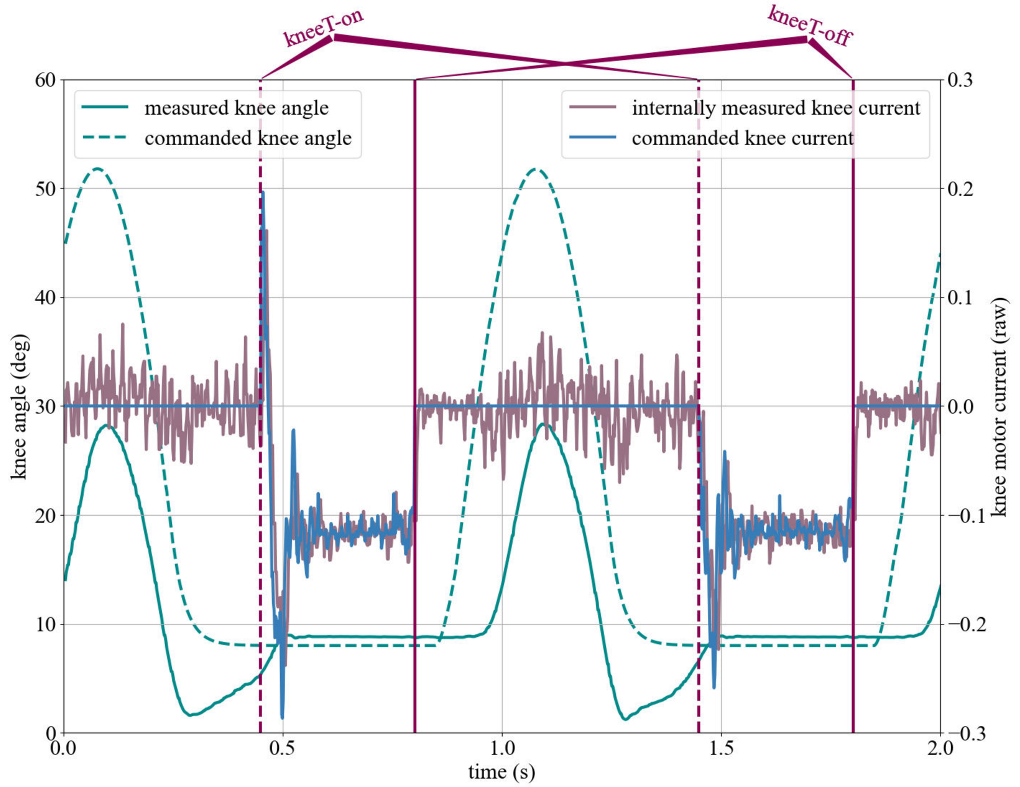

Figure S2. Data from robot in-air motion in passive knee flexion initiation (PKFI) mode. Raw commanded and measured knee angle values (in deg), commanded knee current values, and raw, uncalibrated, internally measured knee motor current values. The commanded knee motor current is zero between the kneeT-off (continuous dark pink vertical line) and kneeT-on (dashed dark pink vertical line). In brushless DC (BLDC) motor control, the commanded motor current values defines the commanded motor torque values. While the commanded knee motor torque is zero, there is no active torque between the upper and lower legs. The knee flexes passively as the hip moves the upper leg and with that, the passive knee joint back and forth.

4 Filter settings

Data		Filter order	Cut-off frequency (Hz)
joint angle	ankle	2	80
	knee	3	20
	hip		
	four-bar mechanism		
joint angular velocity	ankle	3	20
	knee		
	hip		
	four-bar mechanism		
external joint current	hip	3	10
	knee		
external joint torque	hip	3	20
	knee		5
four-bar slider connector horizontal position (from two potentiometers)		3	3.5

Table S1. Butterworth filter orders and cut-off frequencies depending on the data and sensor type.

5 Gait event timing data

time in %GC	AKFI	PKFI	human ref
SAPF	46.25 ***	48.77 ***	48.24
SD of SAPF	0.30	0.18	
TO	53.68	54.47	64.00
SD of TO	0.14	0.20	
LLTD	47.87	46.89	50.00
SD of LLTD	0.15	0.19	
SHF	42.94	47.42	53.27
SD of SHF	0.08	0.28	
SKF	39.00 ***	43.67 ***	43.72
SD of SKF	0.26	0.37	
vmin	46.13	45.33	
SD of vmin	0.27	0.24	
vmax	62.23	61.96	
SD of vmax	0.17	0.26	

Table S2. Timing and standard deviation (SD) of the timing of the gait events in gait cycle percentage (%GC) with active knee flexion initiation (AKFI), and with passive knee flexion initiation (PKFI), and by humans[2]. 0 %GC is the touch-down of the trailing leg. *** denote significant difference between AKFI and PKFI experiments with $p < 0.001$ (for SAPF: $p = 1.80e-21$, for SKF: $p = 1.90e-21$).
Abbreviations: SAPF: Start of Ankle Plantar Flexion, TO: Toe-Off, LLTD: Leading Leg Touch-Down, SHF: Start of Hip Flexion, SKF: Start of Knee Flexion, vmin: time of minimum vertical velocity of the Center of Mass (CoM) of the whole robot, vmax: second vertical velocity peak of the CoM after vmin.

6 Toe height plot

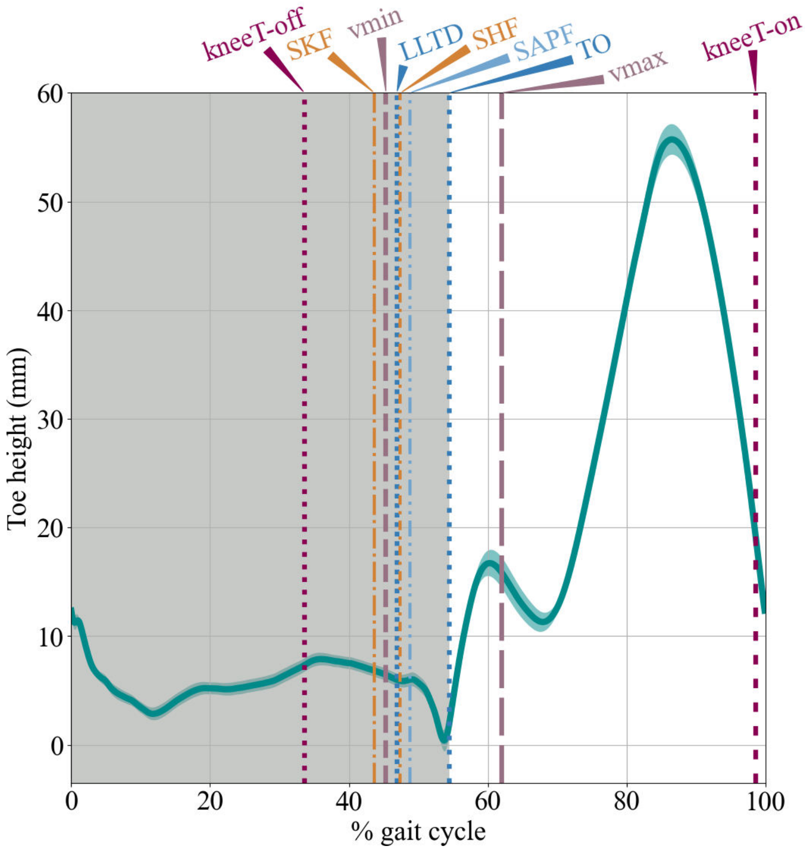

Figure S3. Height of the end of toe segment from the ground in passive knee flexion initiation (PKFI) experiments. Shading shows the standard deviation of the curve. Horizontal axis shows the gait cycle percentage, 0 %GC is the touch-down of the trailing leg. Knee motor torque is zero from the dark pink vertical dotted line (kneeT-off) until the dark pink vertical dashed line (kneeT-on). Gray background during stance phase, white background during swing. Lowest toe height is 2 mm during swing, sufficient for toe clearance.
Abbreviations: SKF: Start of Knee Flexion, SHF: Start of Hip Flexion, SAPF: Start of Ankle Plantar Flexion, vmin: time of minimum vertical velocity of the CoM, LLTD: Leading Leg Touch-Down, TO: Toe-Off, vmax: second vertical velocity peak of the CoM after vmin.

7 Snapshots of the robot gait

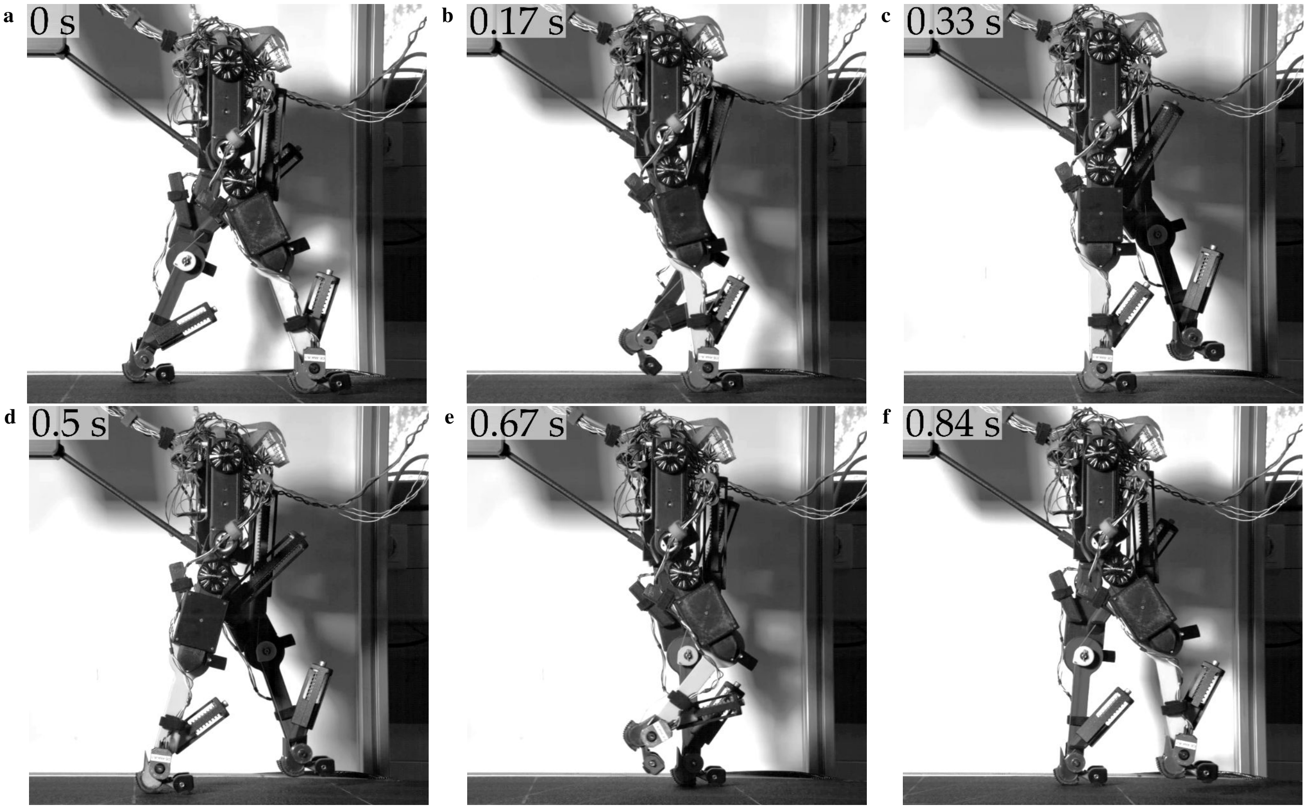

Figure S4. Snapshots of the robot in the passive knee flexion initiation (PKFI) experiment during one gait cycle. One gait cycle lasts 1.0 s. The leg with white lower leg is the trailing leg in this gait cycle example. The robot could swing its leg with a flexed knee with passive knee flexion initiation. Sufficient toe clearance in swing (minimum 2 mm) can be observed in the ground clearance plot (Fig. S3).

8 Momentum and impulse values

Momentum (p)			vmin		LLTD		vmax	
in kg m/s			AKFI	PKFI	AKFI	PKFI	AKFI	PKFI
CoM	x	$p_{CoM,x}$	0.80	0.76	0.79	0.76	0.92	0.93
		SD of $p_{CoM,x}$	0.02	0.02	0.02	0.02	0.02	0.02
	y	$p_{CoM,y}$	-0.48	-0.37	-0.42	-0.31	0.31	0.29
		SD of $p_{CoM,y}$	0.01	0.01	0.01	0.01	0.01	0.01
TL	x	$p_{TL,x}$	0.35	0.23	0.37	0.24	0.52	0.54
		SD of $p_{TL,x}$	0.01	0.01	0.01	0.01	0.01	0.01
	y	$p_{TL,y}$	-0.08	-0.08	-0.06	-0.06	0.15	0.13
		SD of $p_{TL,y}$	0.00	0.00	0.00	0.00	0.00	0.00
RB	x	$p_{RB,x}$	0.44	0.53	0.42	0.52	0.40	0.38
		SD of $p_{RB,x}$	0.02	0.02	0.01	0.01	0.01	0.02
	y	$p_{RB,y}$	-0.39	-0.29	-0.36	-0.25	0.16	0.16
		SD of $p_{RB,y}$	0.01	0.01	0.01	0.01	0.01	0.00

Table S3. Momentum values and their standard deviations (SD) at the beginning (vmin) and end (vmax) of the step-to-step transition period, and at leading leg touch-down (LLTD) in the active knee flexion initiation (AKFI) and the passive knee flexion initiation (PKFI) experiments. **Abbreviations:** x: horizontal direction, y: vertical direction, TL: Trailing Leg, RB: Remaining Body, CoM: Center of Mass of the whole robot.

9 Horizontal and vertical momentum curves

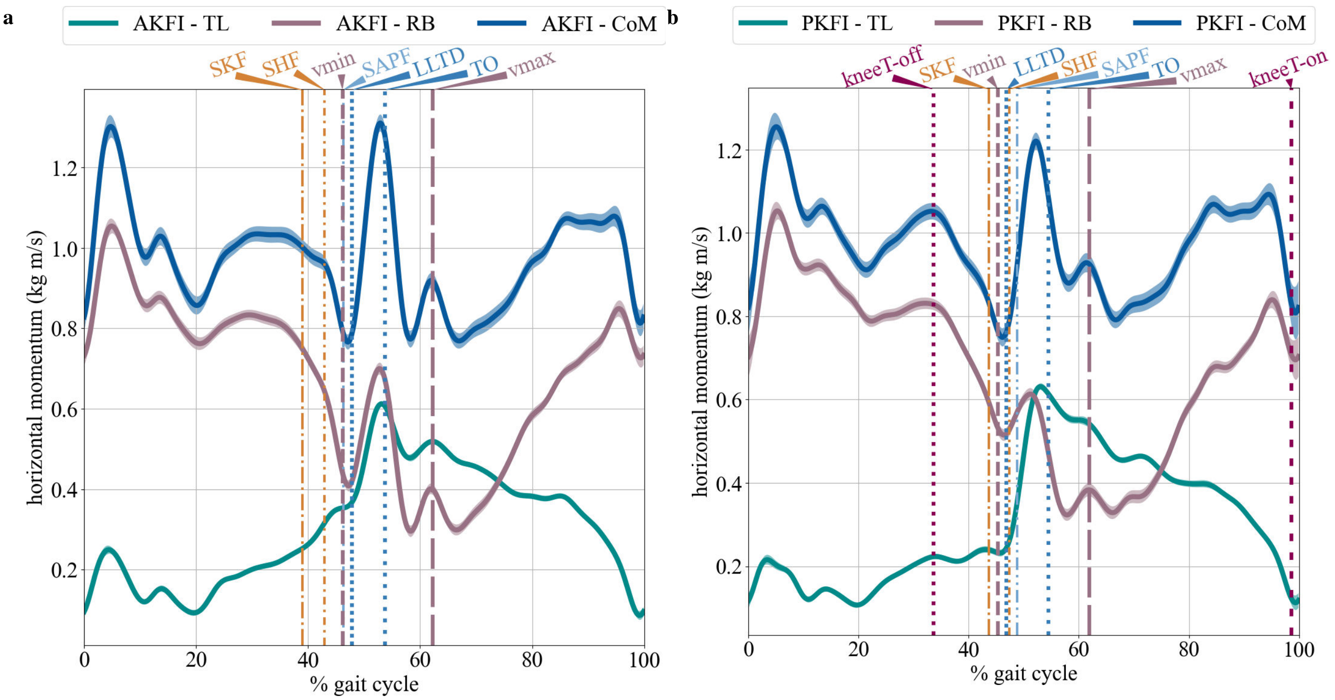

Figure S5. Horizontal momentum of the Remaining Body (RB), Trailing Leg (TL), and Center of Mass (CoM) of the EcoWalker robot during the full gait cycle in the active knee flexion initiation (AKFI - **a**), and in the passive knee flexion initiation (PKFI - **b**) experiments. Shading shows the standard deviation of the curves. Horizontal axis shows the gait cycle percentage, 0 %GC is the touch-down of the trailing leg. In the PKFI experiments, the knee motor torque is zero from the dark pink vertical dotted line (kneeT-off) until the dark pink vertical dashed line (kneeT-on).
Abbreviations: SKF: Start of Knee Flexion, SHF: Start of Hip Flexion, SAPF: Start of Ankle Plantar Flexion, vmin: time of minimum vertical velocity of the CoM, LLTD: Leading Leg Touch-Down, TO: Toe-Off, vmax: second vertical velocity peak of the CoM after vmin.

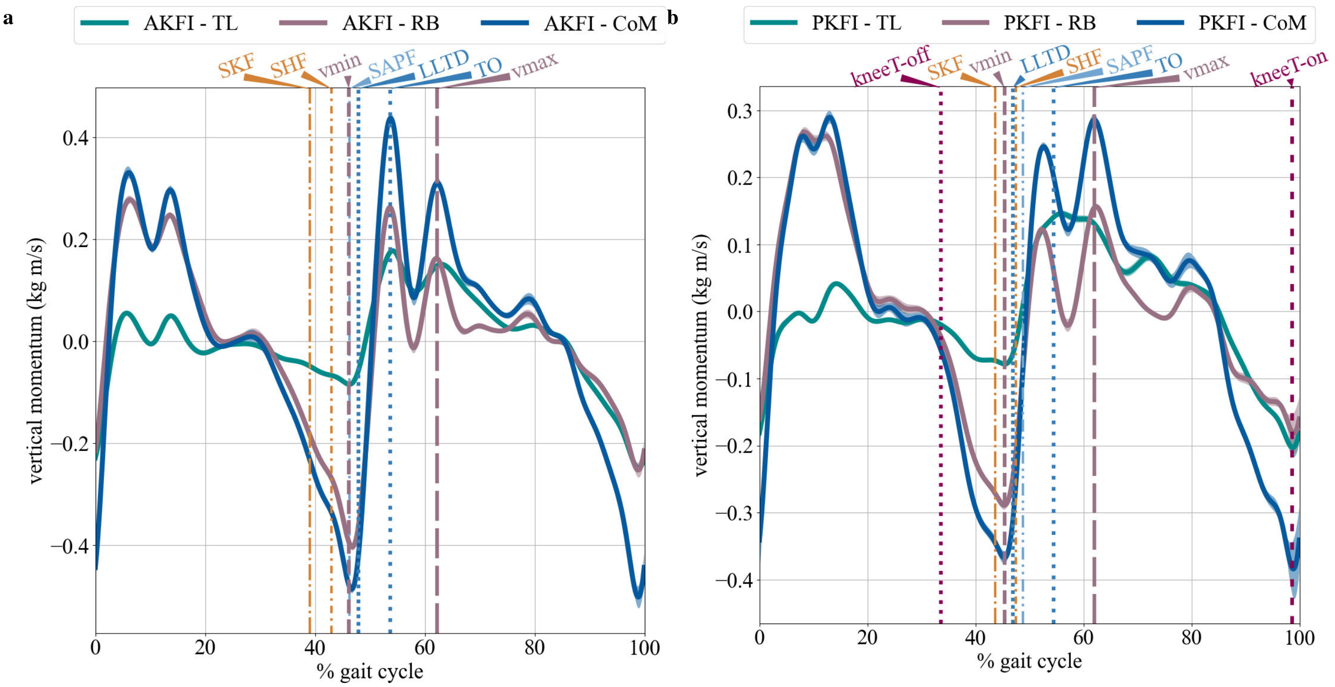

Figure S6. Vertical momentum of the Remaining Body (RB), Trailing Leg (TL), and Center of Mass (CoM) of the EcoWalker robot during the full gait cycle in the active knee flexion initiation (AKFI - **a**), and in the passive knee flexion initiation (PKFI - **b**) experiments. Shading shows the standard deviation of the curves. Horizontal axis shows the gait cycle percentage, 0 %GC is the touch-down of the trailing leg. In the PKFI experiments, the knee motor torque is zero from the dark pink vertical dotted line (kneeT-off) until the dark pink vertical dashed line (kneeT-on).
Abbreviations: SKF: Start of Knee Flexion, SHF: Start of Hip Flexion, SAPF: Start of Ankle Plantar Flexion, vmin: time of minimum vertical velocity of the CoM, LLTD: Leading Leg Touch-Down, TO: Toe-Off, vmax: second vertical velocity peak of the CoM after vmin.

10 Center of mass velocity vector angles

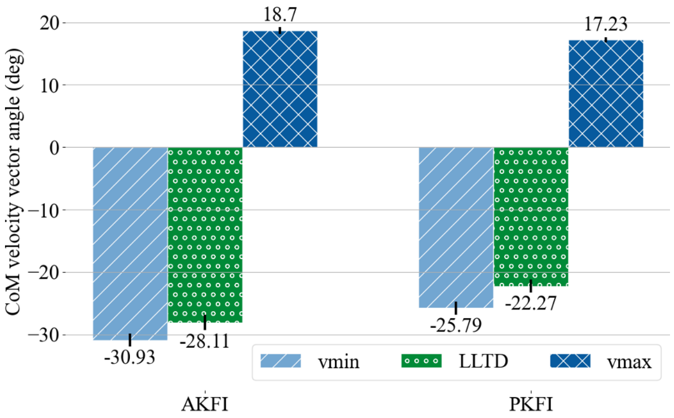

Figure S7. Center of mass velocity vector angles at the start of the step-to-step transition (vmin), at leading leg touch-down (LLTD), and at the end of the step-to-step transition (vmax) in the active knee flexion initiation (AKFI) and in the passive knee flexion initiation (PKFI) experiments. The vertical black lines at the top of the bars show the standard deviations. The velocity vector angle change during the step-to-step transition is larger with AKFI than with PKFI.

11 Center of mass velocity values

CoM velocity (v) in m/s			AKFI	PKFI
vmin	x	$v_{x,vmin}$	0.36	0.34
		SD of $v_{x,vmin}$	0.01	0.01
	y	$v_{y,vmin}$	-0.21	-0.16
		SD of $v_{y,vmin}$	0.00	0.00
LLTD	x	$v_{x,LLTD}$	0.35	0.34
		SD of $v_{x,LLTD}$	0.01	0.01
	y	$v_{y,LLTD}$	-0.19	-0.14
		SD of $v_{y,LLTD}$	0.01	0.01
vmax	x	$v_{x,vmax}$	0.41	0.41
		SD of $v_{x,vmax}$	0.01	0.01
	y	$v_{y,vmax}$	0.14	0.13
		SD of $v_{y,vmax}$	0.00	0.00

Table S4. Center of mass velocity values and their standard deviations (SD) at the beginning (vmin) and end (vmax) of the step-to-step transition period, and at leading leg touch-down (LLTD). **Abbreviations:** x: horizontal direction, y: vertical direction, CoM: Center of Mass of the whole robot.

12 Center of mass total kinetic energy

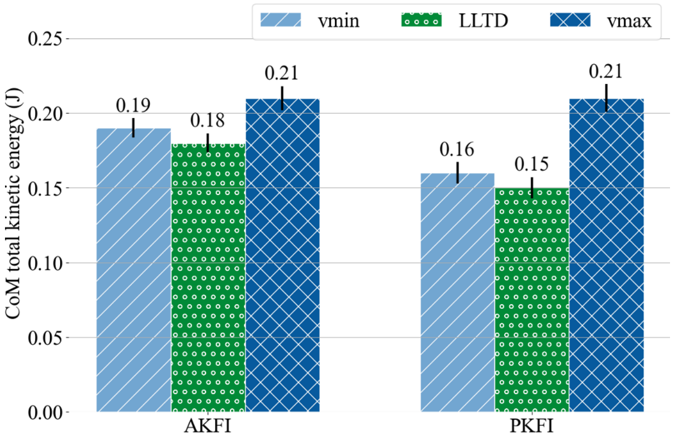

Figure S8. Center of mass total kinetic energy at the start of the step-to-step transition (vmin), at leading leg touch-down (LLTD), and at the end of the step-to-step transition (vmax) in the active knee flexion initiation (AKFI) and in the passive knee flexion initiation (PKFI) experiments. The vertical black lines at the top of the bars show the standard deviations.

13 Center of mass total kinetic energy values

CoM total kinetic energy ($E_{k,CoM,tot}$)		AKFI	PKFI
vmin	$E_{k,CoM,tot,vmin}$	0.19	0.16
	SD of $E_{k,CoM,tot,vmin}$	0.01	0.01
LLTD	$E_{k,CoM,tot,LLTD}$	0.18	0.15
	SD of $E_{k,CoM,tot,LLTD}$	0.01	0.01
vmax	$E_{k,CoM,tot,vmax}$	0.21	0.21
	SD of $E_{k,CoM,tot,vmax}$	0.01	0.01

Table S5. Center of mass (CoM) total kinetic energy values and their standard deviations (SD) at the beginning (vmin) and end (vmax) of the step-to-step transition period, and at leading leg touch-down (LLTD).

14 Ankle power curves

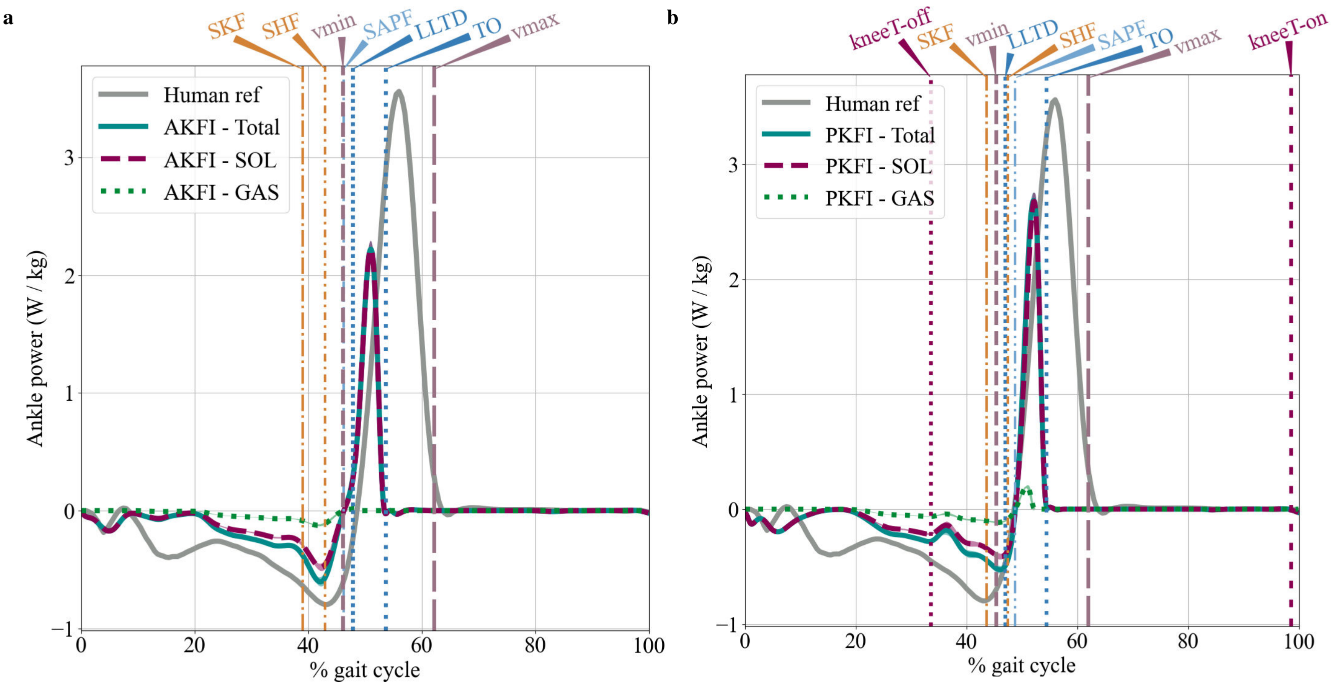

Figure S9. The EcoWalker robot's ankle joint power (sum of SOL and GAS power), SOL, and GAS power contributions during the full gait cycle in the active knee flexion initiation (AKFI - **a**), and in the passive knee flexion initiation (PKFI - **b**) experiments. All powers are in W/kg. Shading shows the standard deviation of the curves. Horizontal axis shows the gait cycle percentage, 0 %GC is the touch-down of the trailing leg. In the PKFI experiments, the knee motor torque is zero from the dark pink vertical dotted line (kneeT-off) until the dark pink vertical dashed line (kneeT-on). The continuous cyan line shows the total ankle power, the dashed dark pink line shows the Soleus (SOL) spring-tendon's contribution to the ankle power, and the dotted green line shows the Gastrocnemius (GAS) spring-tendon's contribution to the ankle power. Ankle power values of human walking are overlayed (gray lines) for reference[2]. Part of ankle unloading is before leading leg touch-down with AKFI, while no unloading happens before leading leg touch-down with PKFI.
Abbreviations: SKF: Start of Knee Flexion, SHF: Start of Hip Flexion, SAPF: Start of Ankle Plantar Flexion, vmin: time of minimum vertical velocity of the CoM, LLTD: Leading Leg Touch-Down, TO: Toe-Off, vmax: second vertical velocity peak of the CoM after vmin.

15 Gait event measurement accuracy in robot and human

gait event	population	SD (in %GC)		
		mean	min	max
LLTD	healthy human	1.71	0.14	6.62
	post stroke human	3.2	0.34	10.2
	EcoWalker robot	0.17	0.14	0.19
TO	healthy human	1.54	0.13	18.23
	post stroke human	3.37	0.31	26.33
	EcoWalker robot	0.17	0.14	0.20

Table S6. Mean, minimum and maximum Standard Deviation (SD) of Leading Leg Touch-Down (LLTD) and Toe-Off (TO) gait event timing data in % of the gait cycle (GC) length. Calculated from the separate SD values of the left leg data and right leg data for each of the 138 healthy adults (healthy human)[3], and from the separate SD values of the paretic leg data and the non-paretic leg data of each of the 50 post stroke adults (post stroke human)[3], and from the separate SD values of the left leg data and the right leg data of two 2-minute experiments (AKFI: active knee flexion initiation and PKFI: passive knee flexion initiation) of the EcoWalker robot.

16 Supplementary Information S2. Right leg result plots

16.1 Joint angles

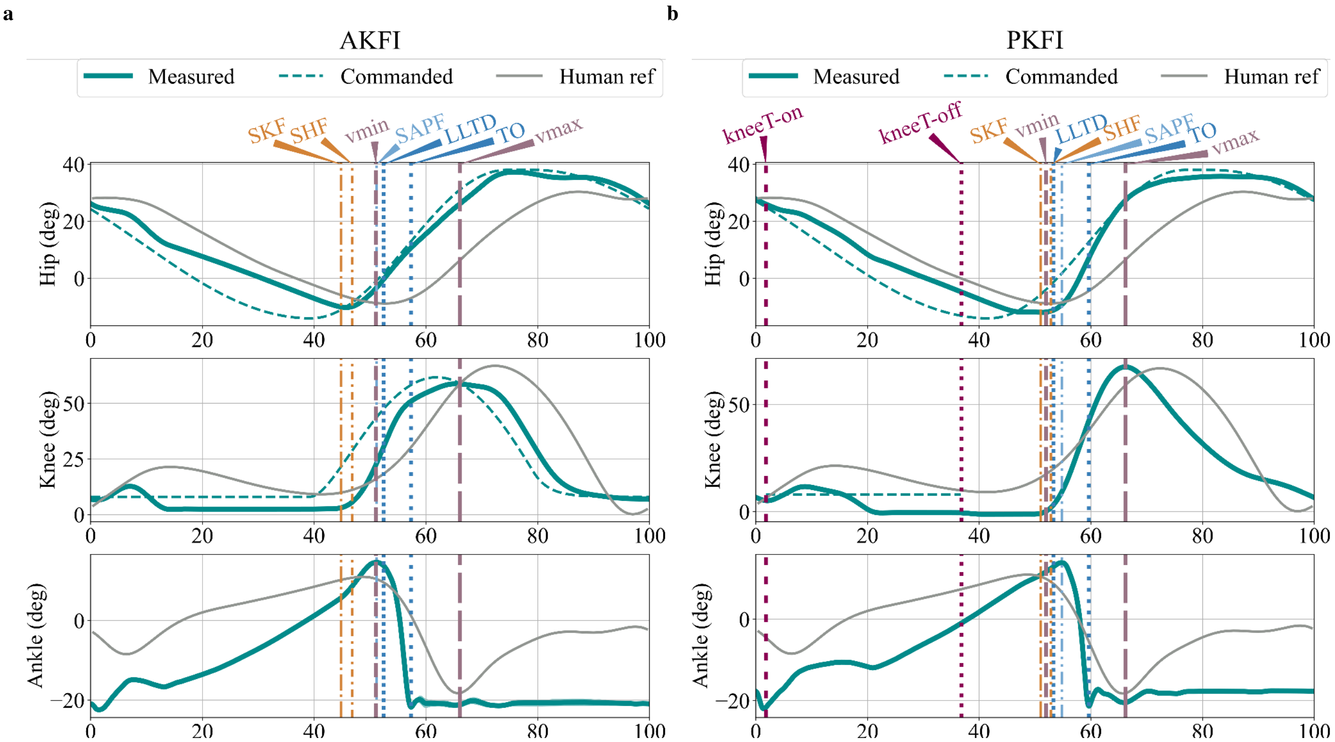

Figure S10. Hip, knee, and ankle angles during the full gait cycle in experiments with active knee flexion initiation (AKFI - **a**), and with passive knee flexion initiation (PKFI - **b**). Shading shows the standard deviation of the curves. Horizontal axis shows the gait cycle percentage, 0 %GC is the touch-down of the trailing leg. In the PKFI experiments, the knee motor torque is zero from the dark pink vertical dotted line (kneeT-off) until the dark pink vertical dashed line (kneeT-on). The continuous cyan line shows the measured joint angles of the robot, while the dashed cyan line shows the commanded joint angles of the hip and the knee. Joint angles of human walking are overlayed (gray lines) for reference.[2: average of trials 20, 21, and 22]

Abbreviations: SKF: Start of Knee Flexion, SHF: Start of Hip Flexion, SAPF: Start of Ankle Plantar Flexion, vmin: time of minimum vertical velocity of the CoM, LLTD: Leading Leg Touch-Down, TO: Toe-Off, vmax: second vertical velocity peak of the CoM after vmin.

16.2 Gait event timings

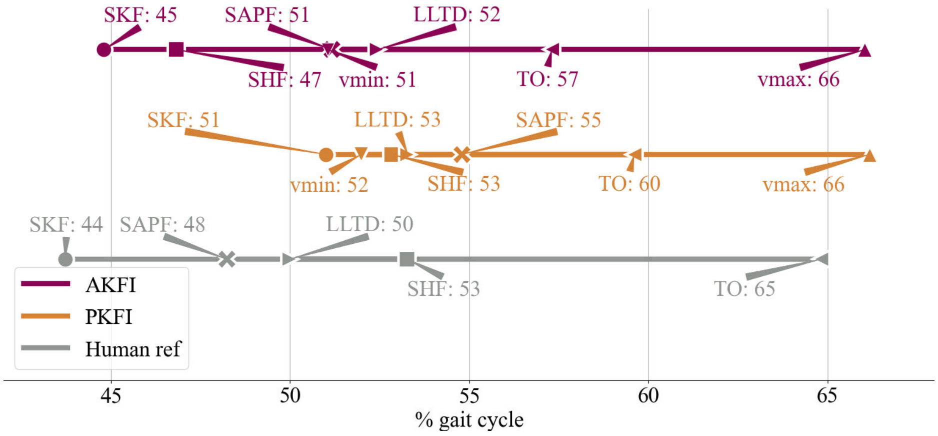

Figure S11. Timing of the gait events in gait cycle percentage with active knee flexion initiation (AKFI), and with passive knee flexion initiation (PKFI), and by humans[2]. 0 %GC is the touch-down of the trailing leg. In the PKFI experiments, knee and hip flexion start 5 %GC later than in the AKFI experiments (SKF and SHF). LLTD occurs 1 %GC earlier with PKFI than with AKFI. The ankle starts to plantarflex (SAPF) 2 %GC after LLTD with PKFI, while SAPF occurs 2 %GC before LLTD with AKFI. The gait event timing values and their standard deviation values are available in Supplementary Table S2.
Abbreviations: ● SKF: Start of Knee Flexion, ■ SHF: Start of Hip Flexion, ✖SAPF: Start of Ankle Plantar Flexion, ▼ vmin: time of minimum vertical velocity of the CoM, ▶ LLTD: Leading Leg Touch-Down, ◀ TO: Toe-Off, ▲ vmax: second vertical velocity peak of the CoM after vmin.

16.3 Statistical test results

	measure	p value	AKFI		PKFI		diff. in %
			mean	SD	mean	SD	
gait events	t_{SKF} (in %GC)	1.83e-21	44.80	0.15	51.00	0.21	13.83
	t_{SAPF} (in %GC)	1.75e-21	51.17	0.14	54.79	0.20	7.08
	$\Delta t_{SAPF-LLTD}$ (in %GC)	1.78e-21	1.27	0.13	-1.50	0.10	-218.15
TL imp.	$\Delta\lvert\mathbf{p}_{TL}\rvert$ (in kg m/s)	1.97e-21	0.14	0.01	0.28	0.01	95.37
	$\Delta p_{TL,x}$ (in kg m/s)	1.97e-21	0.13	0.01	0.27	0.01	105.58
	$\Delta p_{TL,y}$ (in kg m/s)	1.48e-18	0.22	0.00	0.24	0.01	4.96
RB imp.	$\Delta\lvert\mathbf{p}_{RB}\rvert$ (in kg m/s)	0.04	-0.08	0.02	-0.09	0.02	8.63
	$\Delta p_{RB,x}$ (in kg m/s)	1.97e-21	0.06	0.03	-0.03	0.02	-151.11
	$\Delta p_{RB,y}$ (in kg m/s)	1.97e-21	0.56	0.01	0.42	0.01	-23.95
CoM imp.	$\Delta\lvert\mathbf{p}_{CoM}\rvert$ (in kg m/s)	3.69e-21	0.10	0.03	0.20	0.04	102.09
	$\Delta p_{CoM,x}$ (in kg m/s)	3.48e-15	0.19	0.04	0.24	0.04	28.95
	$\Delta p_{CoM,y}$ (in kg m/s)	1.97e-21	0.78	0.01	0.66	0.01	-15.66

Table S7. Results of the Wilcoxon signed-rank tests (p values) to test the differences between the active knee flexion initiation (AKFI) and the passive knee flexion initiation (PKFI) experiments. The tested measures were: time of the start of knee flexion (t_{SKF}), time of the start of ankle plantar flexion (t_{SAPF}), the time period between the start of ankle plantar flexion and the leading leg touch-down ($\Delta t_{SAPF-LLTD}$), the absolute ($\Delta\lvert\mathbf{p}_{TL}\rvert$), horizontal ($\Delta p_{TL,x}$), and vertical ($\Delta p_{TL,y}$) momentum change (impulse) of the trailing leg during the step-to-step transition, the the absolute ($\Delta\lvert\mathbf{p}_{RB}\rvert$), horizontal ($\Delta p_{RB,x}$), and vertical ($\Delta p_{RB,y}$) momentum change (impulse) of the remaining body during the step-to-step transition, and the the absolute ($\Delta\lvert\mathbf{p}_{CoM}\rvert$), horizontal ($\Delta p_{CoM,x}$), and vertical ($\Delta p_{CoM,y}$) momentum change (impulse) of the center of mass during the step-to-step transition. All tested differences were significant with a significance level of $p < 0.05$ or $p < 0.001$. **Abbreviations:** x: horizontal direction, y: vertical direction, TL: Trailing Leg, RB: Remaining Body, CoM: Center of Mass of the whole robot, imp.: impulse = change in momentum, SD: standard deviation, diff. in % was calculated as: $\frac{mean_{PKFI}-mean_{AKFI}}{mean_{AKFI}} \cdot 100$.

16.4 Momentum plots

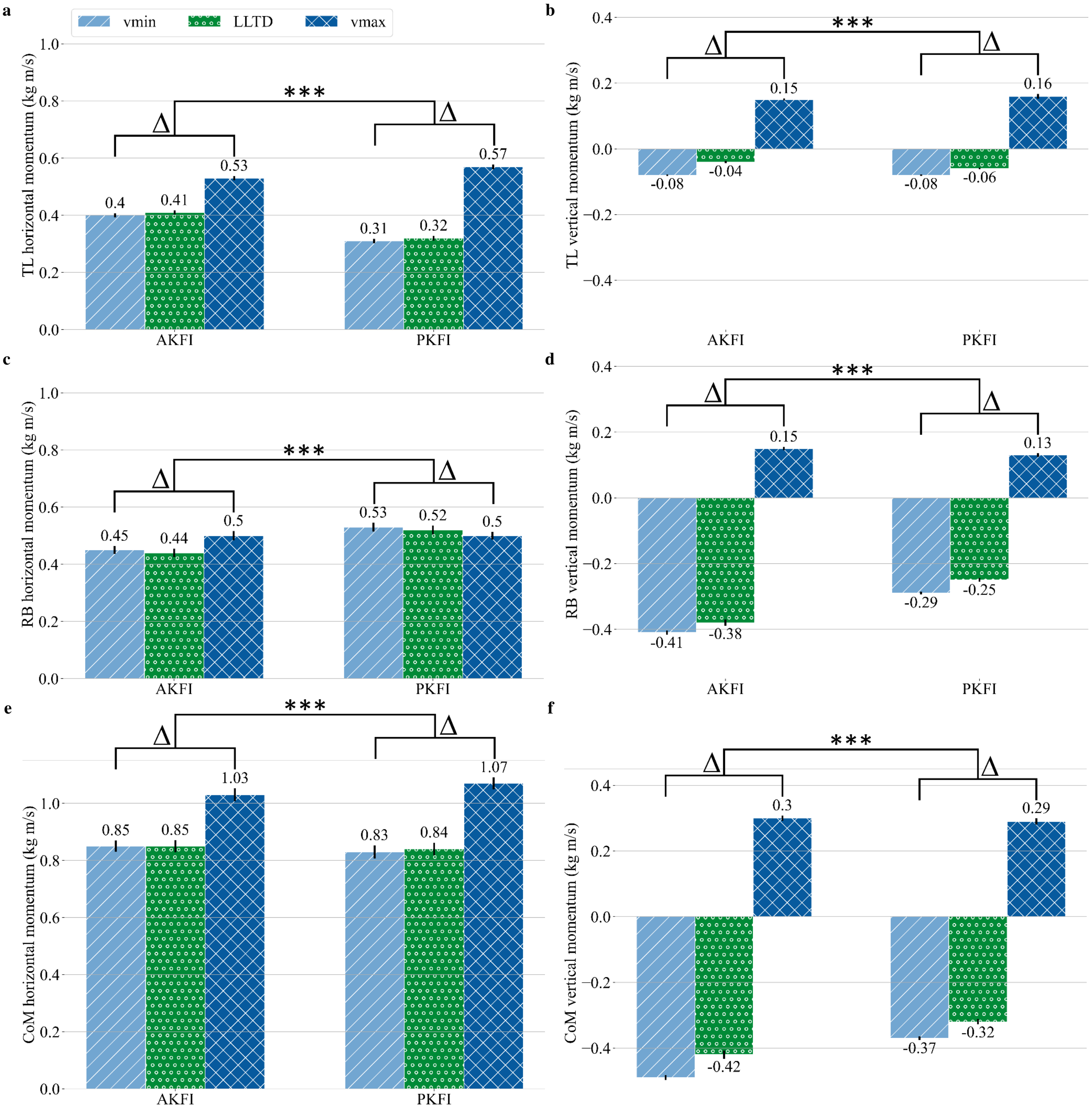

Figure S12. Trailing Leg (TL), Remaining Body (RB), and Center of Mass (CoM) instantaneous momentums in horizontal (**a**, **c**, and **e**) and vertical (**b**, **d**, and **f**) directions at the start of the step-to-step transition (vmin), at leading leg touch-down (LLTD), and at the end of the step-to-step transition (vmax) in the active knee flexion initiation (AKFI) and in the passive knee flexion initiation (PKFI) experiments. The vertical black lines at the top of the bars show the standard deviations. The change in TL horizontal momentum is larger with PKFI than with AKFI, and RB horizontal momentum decreases with PKFI. The CoM's vertical momentum increases more in AKFI than in PKFI experiments during the step-to-step transition. The momentum values and their standard deviation values are available in Supplementary Table S3. *** denote significant difference between the momentum changes (Δ) during the step-to-step transition period in the AKFI and in the PKFI experiments with $p < 0.001$. The exact p values are available in Tab. S7.

16.5 Center of mass velocity vectors

a b

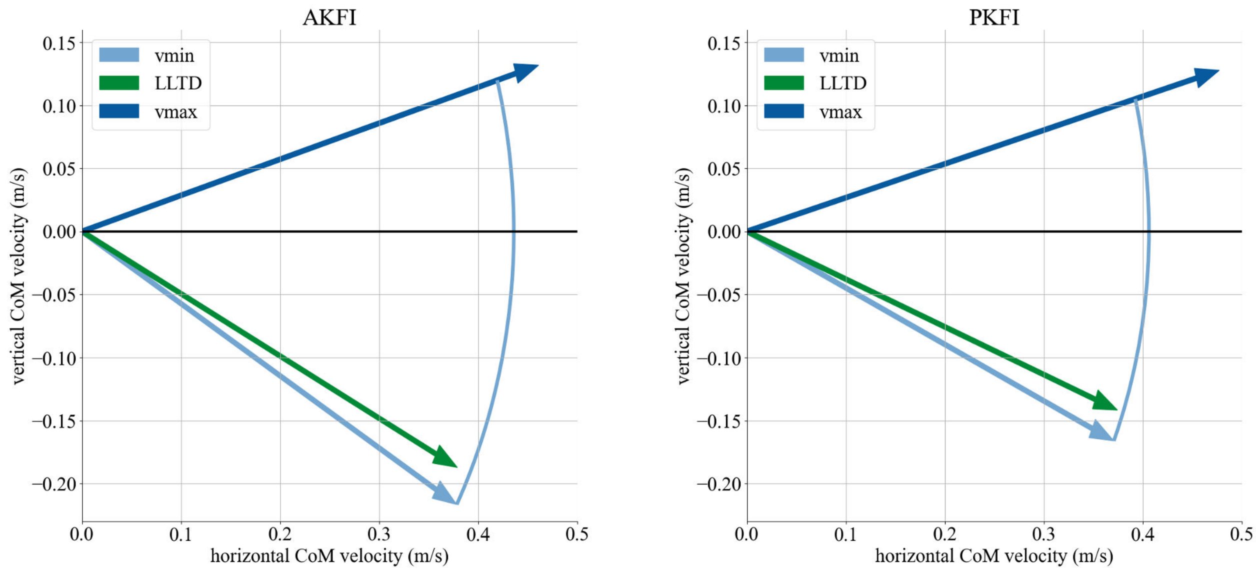

Figure S13. Center of Mass (CoM) velocity vectors at the start of the step-to-step transition (vmin), at leading leg touch-down (LLTD), and at the end of the step-to-step transition (vmax) in the active knee flexion initiation (AKFI - **a**) and in the passive knee flexion initiation (PKFI - **b**) experiments. An arc is drawn with a radius that is equal to the length of the velocity vector at vmin to better show the relation of the vector lengths at the three different times during the step-to-step transition. The length of the velocity vector increases between vmin and vmax more in the PKFI experiments than in the AKFI experiments.

17 Supplementary Information S3. PKFI40 result plots

17.1 Left Leg as Trailing Leg

17.1.1 Joint angles

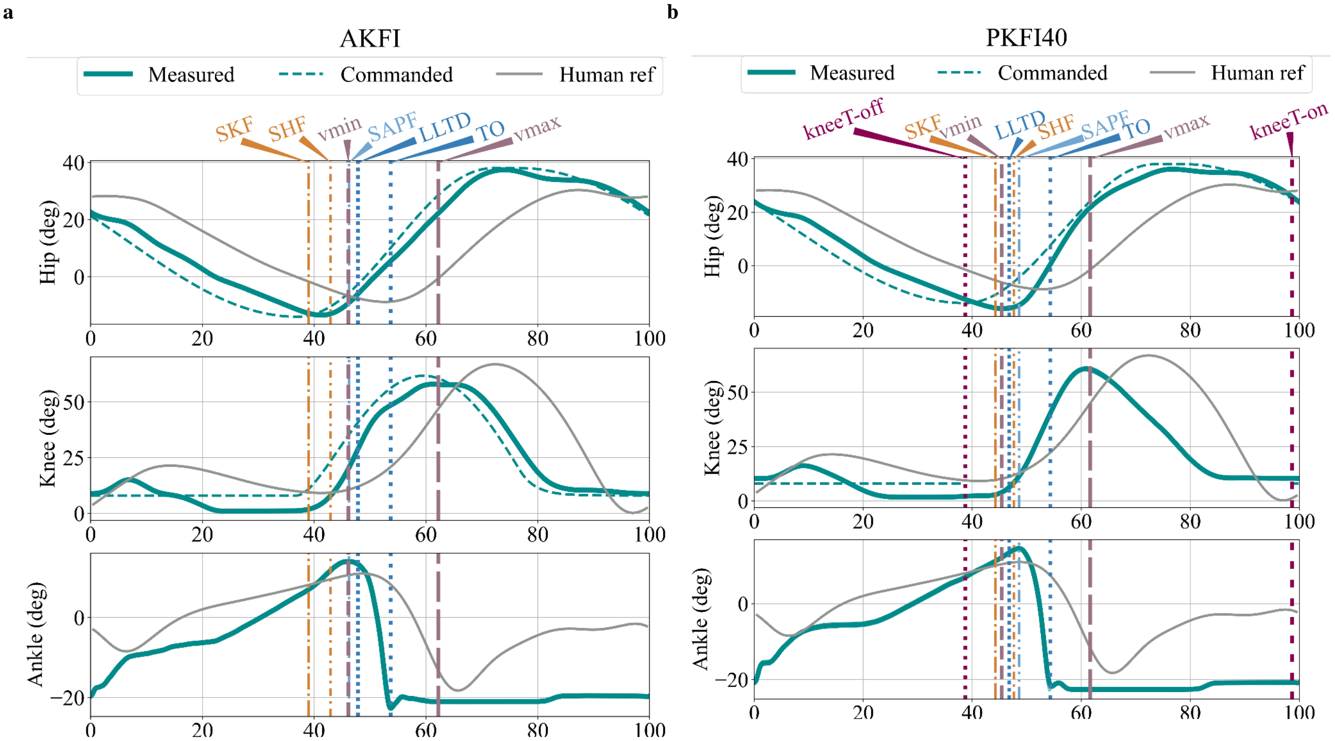

Figure S14. Hip, knee, and ankle angles during the full gait cycle in experiments with active knee flexion initiation (AKFI - **a**), and with passive knee flexion initiation (PKFI - **b**). Shading shows the standard deviation of the curves. Horizontal axis shows the gait cycle percentage, 0 %GC is the touch-down of the trailing leg. In the PKFI experiments, the knee motor torque is zero from the dark pink vertical dotted line (kneeT-off) until the dark pink vertical dashed line (kneeT-on). The continuous cyan line shows the measured joint angles of the robot, while the dashed cyan line shows the commanded joint angles of the hip and the knee. Joint angles of human walking are overlayed (gray lines) for reference.[2]: average of trials 20, 21, and 22

Abbreviations: SKF: Start of Knee Flexion, SHF: Start of Hip Flexion, SAPF: Start of Ankle Plantar Flexion, vmin: time of minimum vertical velocity of the CoM, LLTD: Leading Leg Touch-Down, TO: Toe-Off, vmax: second vertical velocity peak of the CoM after vmin.

17.1.2 Gait event timings

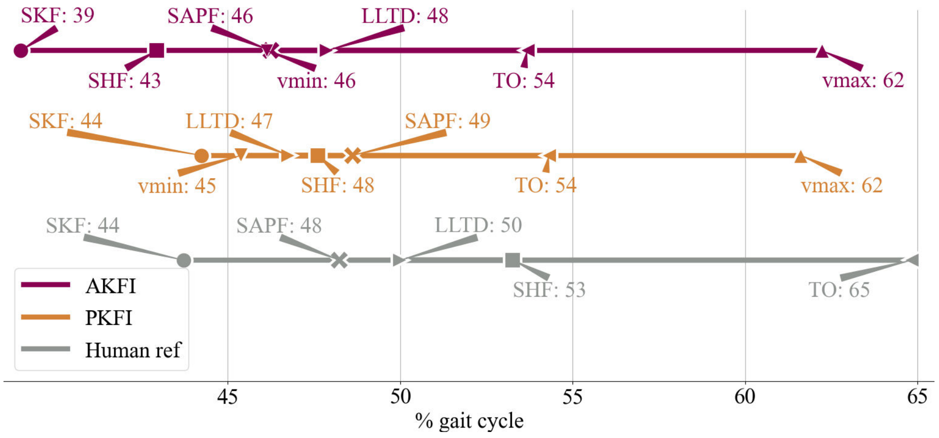

Figure S15. Timing of the gait events in gait cycle percentage with active knee flexion initiation (AKFI), and with passive knee flexion initiation (PKFI), and by humans[2]. 0 %GC is the touch-down of the trailing leg. In the PKFI experiments, knee and hip flexion start 5 %GC later than in the AKFI experiments (SKF and SHF). LLTD occurs 1 %GC earlier with PKFI than with AKFI. The ankle starts to plantarflex (SAPF) 2 %GC after LLTD with PKFI, while SAPF occurs 2 %GC before LLTD with AKFI. The gait event timing values and their standard deviation values are available in Supplementary Table S2.
Abbreviations: ● SKF: Start of Knee Flexion, ■ SHF: Start of Hip Flexion, ✖SAPF: Start of Ankle Plantar Flexion, ▼ vmin: time of minimum vertical velocity of the CoM, ▶ LLTD: Leading Leg Touch-Down, ◀ TO: Toe-Off, ▲ vmax: second vertical velocity peak of the CoM after vmin.

17.1.3 Statistical test results

	measure	p value	AKFI		PKFI		diff. in %
			mean	SD	mean	SD	
gait events	t_{SKF} (in %GC)	1.92E-21	39.00	0.26	44.23	0.52	13.43
	t_{SAPF} (in %GC)	1.86E-21	46.25	0.30	48.62	0.33	5.13
	$\Delta t_{SAPF-LLTD}$ (in %GC)	1.89E-21	1.62	0.33	-1.85	0.11	-213.85
TL imp.	$\Delta\lvert\mathbf{p}_{TL}\rvert$ (in kg m/s)	1.97E-21	0.18	0.01	0.32	0.01	78.43
	$\Delta p_{TL,x}$ (in kg m/s)	1.97E-21	0.17	0.01	0.31	0.01	88.32
	$\Delta p_{TL,y}$ (in kg m/s)	2.02E-21	0.23	0.00	0.21	0.01	-9.99
RB imp.	$\Delta\lvert\mathbf{p}_{RB}\rvert$ (in kg m/s)	1.97E-21	-0.16	0.02	-0.22	0.02	36.12
	$\Delta p_{RB,x}$ (in kg m/s)	1.97E-21	-0.04	0.03	-0.17	0.02	308.59
	$\Delta p_{RB,y}$ (in kg m/s)	1.97E-21	0.55	0.01	0.44	0.01	-21.42
CoM imp.	$\Delta\lvert\mathbf{p}_{CoM}\rvert$ (in kg m/s)	1.68E-17	0.04	0.03	0.10	0.03	129.57
	$\Delta p_{CoM,x}$ (in kg m/s)	3.54E-04	0.12	0.03	0.14	0.04	12.49
	$\Delta p_{CoM,y}$ (in kg m/s)	1.97E-21	0.79	0.01	0.65	0.01	-18.04

Table S8. Results of the Wilcoxon signed-rank tests (p values) to test the differences between the active knee flexion initiation (AKFI) and the passive knee flexion initiation (PKFI) experiments. The tested measures were: time of the start of knee flexion (t_{SKF}), time of the start of ankle plantar flexion (t_{SAPF}), the time period between the start of ankle plantar flexion and the leading leg touch-down ($\Delta t_{SAPF-LLTD}$), the absolute ($\Delta\lvert\mathbf{p}_{TL}\rvert$), horizontal ($\Delta p_{TL,x}$), and vertical ($\Delta p_{TL,y}$) momentum change (impulse) of the trailing leg during the step-to-step transition, the the absolute ($\Delta\lvert\mathbf{p}_{RB}\rvert$), horizontal ($\Delta p_{RB,x}$), and vertical ($\Delta p_{RB,y}$) momentum change (impulse) of the remaining body during the step-to-step transition, and the the absolute ($\Delta\lvert\mathbf{p}_{CoM}\rvert$), horizontal ($\Delta p_{CoM,x}$), and vertical ($\Delta p_{CoM,y}$) momentum change (impulse) of the center of mass during the step-to-step transition. All tested differences were significant with a significance level of $p < 0.001$. **Abbreviations:** x: horizontal direction, y: vertical direction, TL: Trailing Leg, RB: Remaining Body, CoM: Center of Mass of the whole robot, imp.: impulse = change in momentum, SD: standard deviation, diff. in % was calculated as: $\frac{mean_{PKFI} - mean_{AKFI}}{mean_{AKFI}} \cdot 100$.

17.1.4 Momentum plots

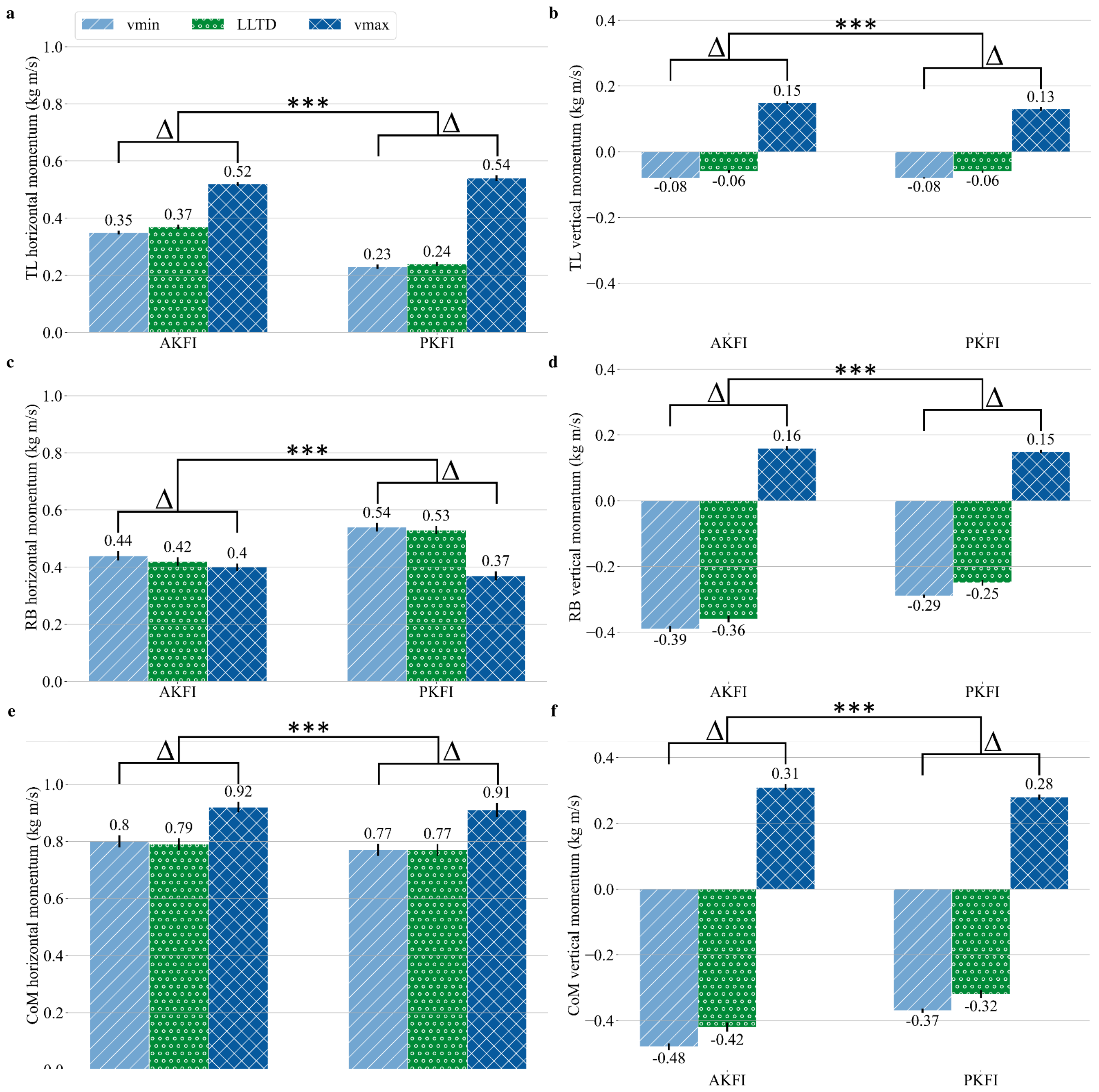

Figure S16. Trailing Leg (TL), Remaining Body (RB), and Center of Mass (CoM) instantaneous momentums in horizontal (**a**, **c**, and **e**) and vertical (**b**, **d**, and **f**) directions at the start of the step-to-step transition (vmin), at leading leg touch-down (LLTD), and at the end of the step-to-step transition (vmax) in the active knee flexion initiation (AKFI) and in the passive knee flexion initiation (PKFI) experiments. The vertical black lines at the top of the bars show the standard deviations. The change in TL horizontal momentum is larger with PKFI than with AKFI, and RB horizontal momentum decreases with PKFI. The CoM's vertical momentum increases more in AKFI than in PKFI experiments during the step-to-step transition. The momentum values and their standard deviation values are available in Supplementary Table S3. *** denote significant difference between the momentum changes (Δ) during the step-to-step transition period in the AKFI and in the PKFI experiments with $p < 0.001$. The exact p values are available in Tab. S8.

17.1.5 Center of mass velocity vectors

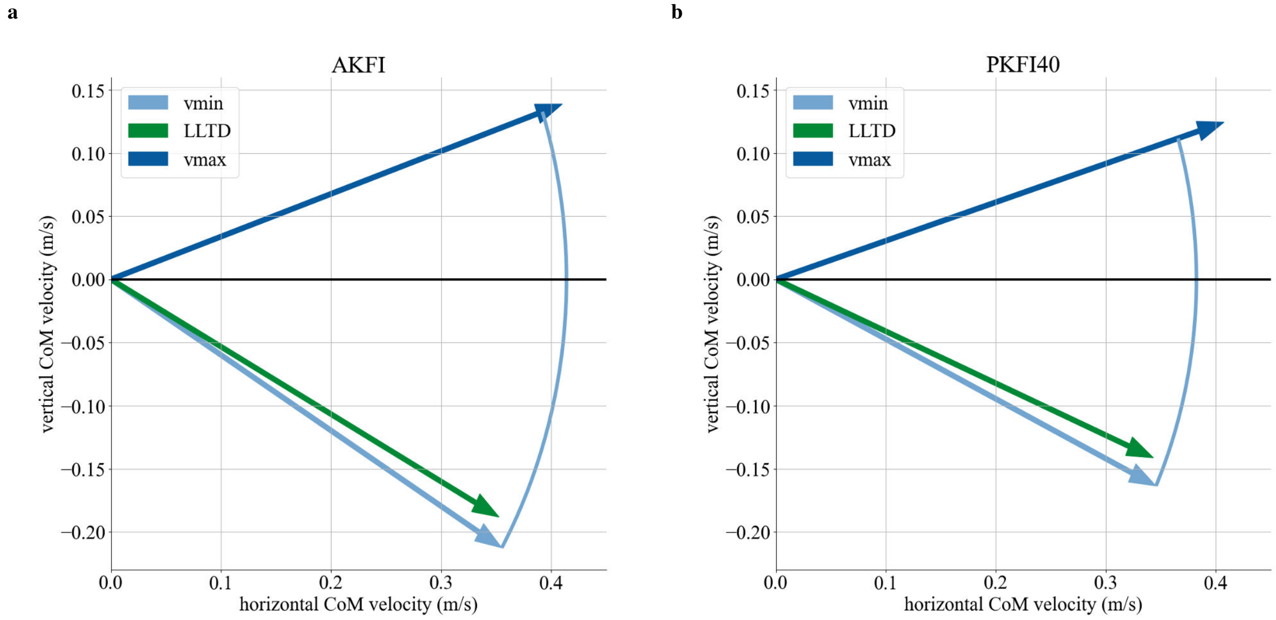

Figure S17. Center of Mass (CoM) velocity vectors at the start of the step-to-step transition (vmin), at leading leg touch-down (LLTD), and at the end of the step-to-step transition (vmax) in the active knee flexion initiation (AKFI - **a**) and in the passive knee flexion initiation (PKFI - **b**) experiments. An arc is drawn with a radius that is equal to the length of the velocity vector at vmin to better show the relation of the vector lengths at the three different times during the step-to-step transition. The length of the velocity vector increases between vmin and vmax more in the PKFI experiments than in the AKFI experiments.

17.2 Right Leg as Trailing Leg

17.2.1 Joint angles

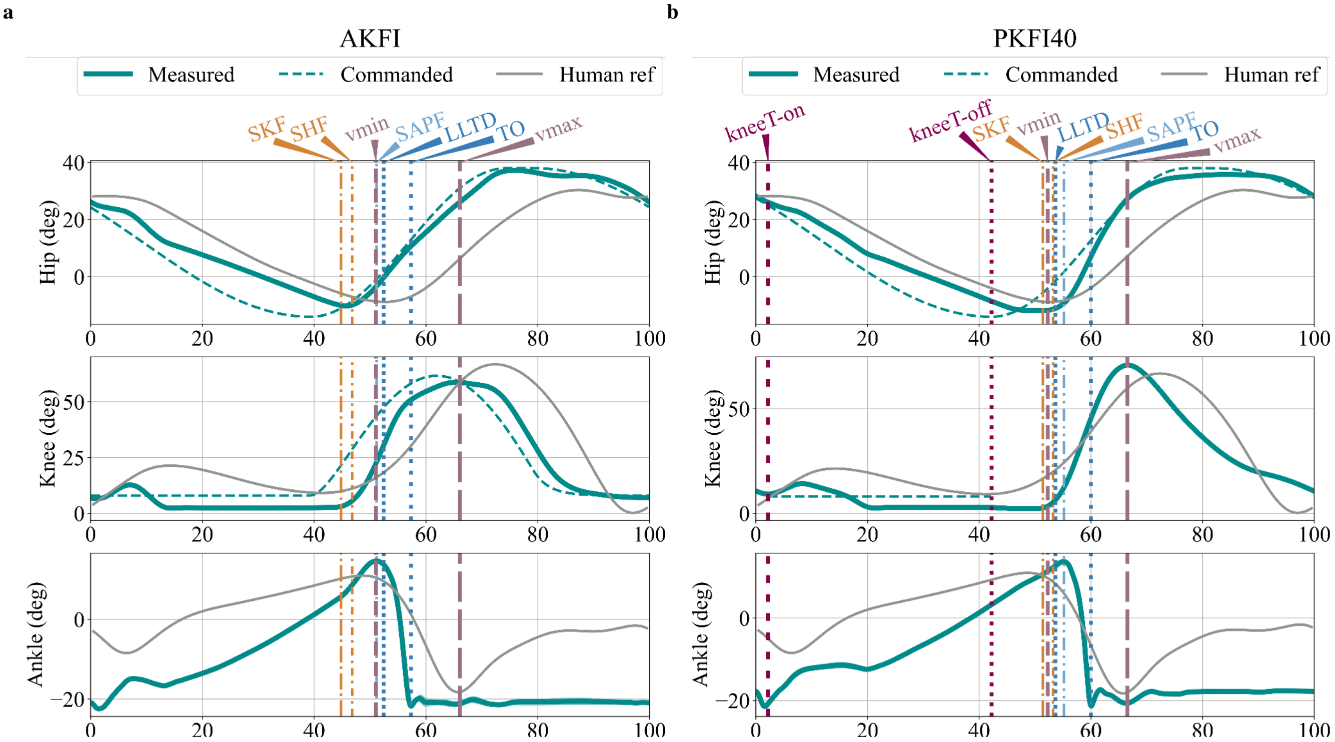

Figure S18. Hip, knee, and ankle angles during the full gait cycle in experiments with active knee flexion initiation (AKFI - **a**), and with passive knee flexion initiation (PKFI - **b**). Shading shows the standard deviation of the curves. Horizontal axis shows the gait cycle percentage, 0 %GC is the touch-down of the trailing leg. In the PKFI experiments, the knee motor torque is zero from the dark pink vertical dotted line (kneeT-off) until the dark pink vertical dashed line (kneeT-on). The continuous cyan line shows the measured joint angles of the robot, while the dashed cyan line shows the commanded joint angles of the hip and the knee. Joint angles of human walking are overlayed (gray lines) for reference.[2]: average of trials 20, 21, and 22

Abbreviations: SKF: Start of Knee Flexion, SHF: Start of Hip Flexion, SAPF: Start of Ankle Plantar Flexion, vmin: time of minimum vertical velocity of the CoM, LLTD: Leading Leg Touch-Down, TO: Toe-Off, vmax: second vertical velocity peak of the CoM after vmin.

17.2.2 Gait event timings

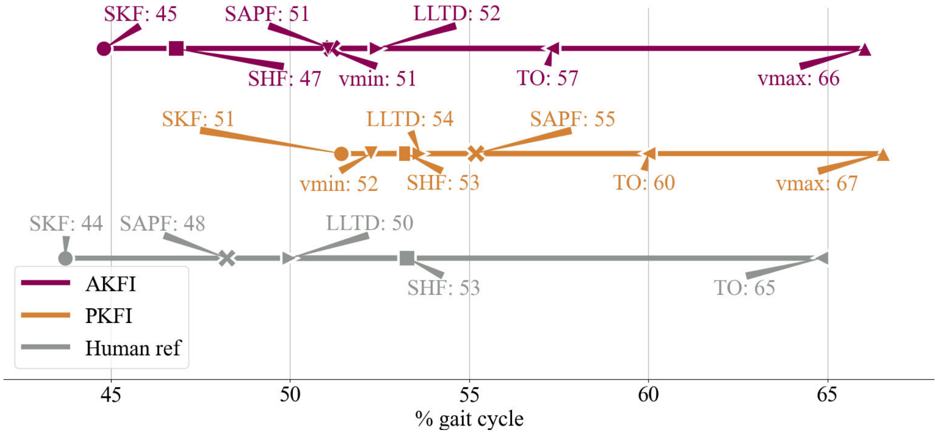

Figure S19. Timing of the gait events in gait cycle percentage with active knee flexion initiation (AKFI), and with passive knee flexion initiation (PKFI), and by humans[2]. 0 %GC is the touch-down of the trailing leg. In the PKFI experiments, knee and hip flexion start 5 %GC later than in the AKFI experiments (SKF and SHF). LLTD occurs 1 %GC earlier with PKFI than with AKFI. The ankle starts to plantarflex (SAPF) 2 %GC after LLTD with PKFI, while SAPF occurs 2 %GC before LLTD with AKFI. The gait event timing values and their standard deviation values are available in Supplementary Table S2. **Abbreviations:** ● SKF: Start of Knee Flexion, ■ SHF: Start of Hip Flexion, ✖SAPF: Start of Ankle Plantar Flexion, ▼ vmin: time of minimum vertical velocity of the CoM, ▶ LLTD: Leading Leg Touch-Down, ◀ TO: Toe-Off, ▲ vmax: second vertical velocity peak of the CoM after vmin.

17.2.3 Statistical test results

	measure	p value	AKFI		PKFI		diff. in %
			mean	SD	mean	SD	
gait events	t_{SKF} (in %GC)	1.84E-21	44.80	0.15	51.42	0.22	14.77
	t_{SAPF} (in %GC)	1.73E-21	51.17	0.14	55.19	0.23	7.86
	$\Delta t_{SAPF-LLTD}$ (in %GC)	1.78E-21	1.27	0.13	-1.56	0.15	-222.28
TL imp.	$\Delta\lvert\mathbf{p}_{TL}\rvert$ (in kg m/s)	1.97E-21	0.14	0.01	0.27	0.01	92.55
	$\Delta p_{TL,x}$ (in kg m/s)	1.97E-21	0.13	0.01	0.26	0.01	102.99
	$\Delta p_{TL,y}$ (in kg m/s)	1.24E-11	0.22	0.00	0.23	0.01	2.31
RB imp.	$\Delta\lvert\mathbf{p}_{RB}\rvert$ (in kg m/s)	0.16	-0.08	0.02	-0.09	0.02	5.31
	$\Delta p_{RB,x}$ (in kg m/s)	2.02E-21	0.06	0.03	-0.02	0.02	-140.30
	$\Delta p_{RB,y}$ (in kg m/s)	1.97E-21	0.56	0.01	0.43	0.01	-23.53
CoM imp.	$\Delta\lvert\mathbf{p}_{CoM}\rvert$ (in kg m/s)	2.13E-21	0.10	0.03	0.20	0.03	101.50
	$\Delta p_{CoM,x}$ (in kg m/s)	2.47E-17	0.19	0.04	0.24	0.03	30.36
	$\Delta p_{CoM,y}$ (in kg m/s)	1.97E-21	0.78	0.01	0.65	0.01	-16.12

Table S9. Results of the Wilcoxon signed-rank tests (p values) to test the differences between the active knee flexion initiation (AKFI) and the passive knee flexion initiation (PKFI) experiments. The tested measures were: time of the start of knee flexion (t_{SKF}), time of the start of ankle plantar flexion (t_{SAPF}), the time period between the start of ankle plantar flexion and the leading leg touch-down ($\Delta t_{SAPF-LLTD}$), the absolute ($\Delta\lvert\mathbf{p}_{TL}\rvert$), horizontal ($\Delta p_{TL,x}$), and vertical ($\Delta p_{TL,y}$) momentum change (impulse) of the trailing leg during the step-to-step transition, the the absolute ($\Delta\lvert\mathbf{p}_{RB}\rvert$), horizontal ($\Delta p_{RB,x}$), and vertical ($\Delta p_{RB,y}$) momentum change (impulse) of the remaining body during the step-to-step transition, and the the absolute ($\Delta\lvert\mathbf{p}_{CoM}\rvert$), horizontal ($\Delta p_{CoM,x}$), and vertical ($\Delta p_{CoM,y}$) momentum change (impulse) of the center of mass during the step-to-step transition. All tested differences were significant with a significance level of $p < 0.001$ except the magnitude change of the remaining body momentum vector. **Abbreviations:** x: horizontal direction, y: vertical direction, TL: Trailing Leg, RB: Remaining Body, CoM: Center of Mass of the whole robot, imp.: impulse = change in momentum, SD: standard deviation, diff. in % was calculated as: $\frac{mean_{PKFI} - mean_{AKFI}}{mean_{AKFI}} \cdot 100$.

17.2.4 Momentum plots

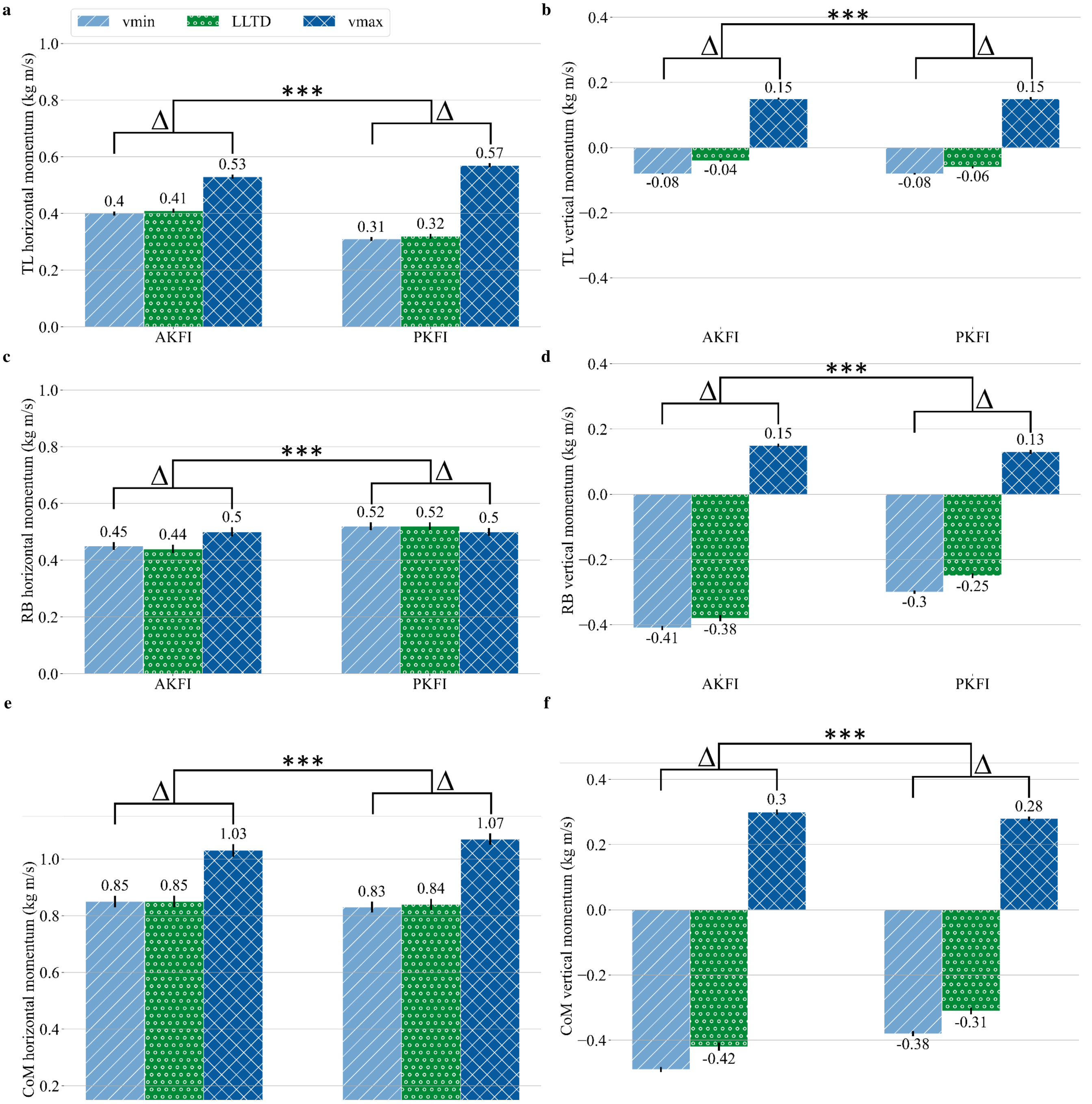

Figure S20. Trailing Leg (TL), Remaining Body (RB), and Center of Mass (CoM) instantaneous momentums in horizontal (**a**, **c**, and **e**) and vertical (**b**, **d**, and **f**) directions at the start of the step-to-step transition (vmin), at leading leg touch-down (LLTD), and at the end of the step-to-step transition (vmax) in the active knee flexion initiation (AKFI) and in the passive knee flexion initiation (PKFI) experiments. The vertical black lines at the top of the bars show the standard deviations. The change in TL horizontal momentum is larger with PKFI than with AKFI, and RB horizontal momentum decreases with PKFI. The CoM's vertical momentum increases more in AKFI than in PKFI experiments during the step-to-step transition. The momentum values and their standard deviation values are available in Supplementary Table S3. *** denote significant difference between the momentum changes (Δ) during the step-to-step transition period in the AKFI and in the PKFI experiments with $p < 0.001$. The exact p values are available in Tab. S9.

17.2.5 Center of mass velocity vectors

a b

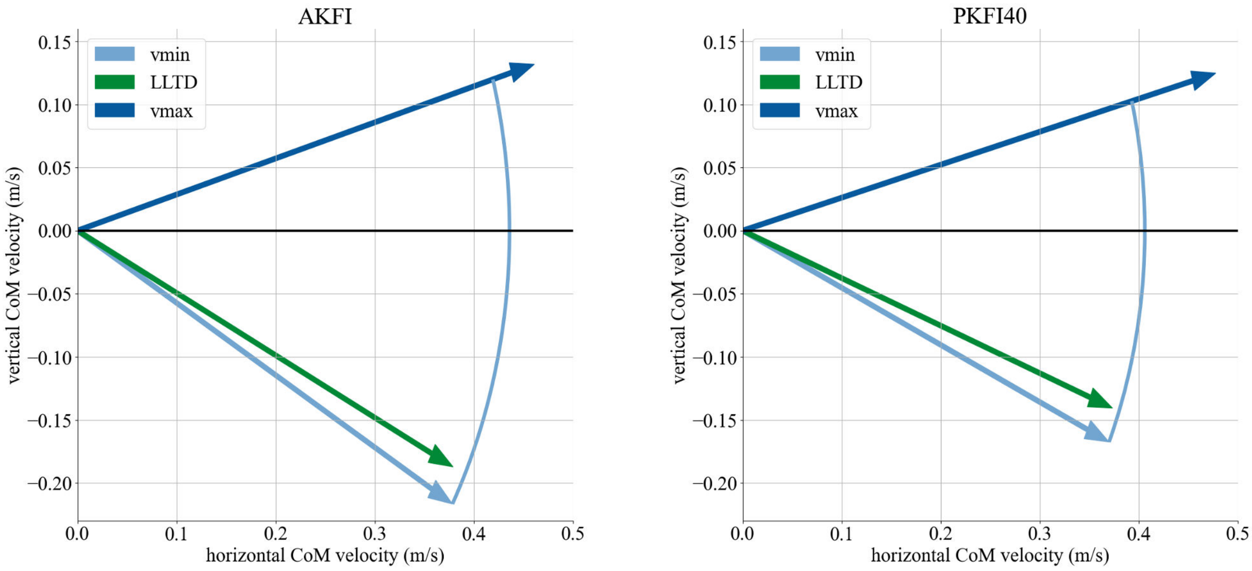

Figure S21. Center of Mass (CoM) velocity vectors at the start of the step-to-step transition (vmin), at leading leg touch-down (LLTD), and at the end of the step-to-step transition (vmax) in the active knee flexion initiation (AKFI - **a**) and in the passive knee flexion initiation (PKFI - **b**) experiments. An arc is drawn with a radius that is equal to the length of the velocity vector at vmin to better show the relation of the vector lengths at the three different times during the step-to-step transition. The length of the velocity vector increases between vmin and vmax more in the PKFI experiments than in the AKFI experiments.

18 Supplementary Videos

Movie S1: Visual summary of the main findings of the paper. After listing the main goals, we list the main results with the corresponding figures. At the end of the video, a high-speed recording of the two experiments is shown side-by-side for easier comparison.

Movie S2: Real-time and high-speed recordings of the different robot gaits during the experiments. First, we show a real-time and a high-speed video recording of the Active Knee Flexion Initiation (AKFI) experiment. Then, we show a real-time and a high-speed video recording of the Passive Knee Flexion Initiation (PKFI) experiment.

References

1. Adamczyk, P. G. & Kuo, A. D. Redirection of center-of-mass velocity during the step-to-step transition of human walking. *J. Exp. Biol.* **212**, 2668–2678, DOI: 10.1242/jeb.027581 (2009).

2. van der Zee, T. J., Mundinger, E. M. & Kuo, A. D. A biomechanics dataset of healthy human walking at various speeds, step lengths and step widths. *Sci. Data* **9**, 704, DOI: 10.1038/s41597-022-01817-1 (2022).

3. Van Criekinge, T. *et al.* A full-body motion capture gait dataset of 138 able-bodied adults across the life span and 50 stroke survivors. *Sci. Data* **10**, 1–8, DOI: 10.1038/s41597-023-02767-y (2023).
